# Author Correction: A RUNX-targeted gene switch-off approach modulates the BIRC5/PIF1-p21 pathway and reduces glioblastoma growth in mice

**DOI:** 10.1038/s42003-022-04006-3

**Published:** 2022-09-27

**Authors:** Etsuko Yamamoto Hattori, Tatsuya Masuda, Yohei Mineharu, Masamitsu Mikami, Yukinori Terada, Yasuzumi Matsui, Hirohito Kubota, Hidemasa Matsuo, Masahiro Hirata, Tatsuki R. Kataoka, Tatsutoshi Nakahata, Shuji Ikeda, Susumu Miyamoto, Hiroshi Sugiyama, Yoshiki Arakawa, Yasuhiko Kamikubo

**Affiliations:** 1 Department of Neurosurgery, Graduate School of Medicine, Kyoto University; Kyoto City, Kyoto, 606-8507 Japan; 2 Department of Human Health Sciences, Graduate School of Medicine, Kyoto University; Kyoto City, Kyoto, 606-8507 Japan; 3 Department of Pediatrics, Graduate School of Medicine, Kyoto University; Kyoto City, Kyoto, 606-8507 Japan; 4 Department of Diagnostic Pathology, Kyoto University Hospital; Kyoto City, Kyoto, 606-8507 Japan; 5 Drug Discovery Technology Development Office, Center for iPS Cell Research and Application (CiRA), Kyoto University; Kyoto City, Kyoto, 606-8507 Japan; 6 Department of Chemistry, Graduate School of Science, Kyoto University; Kyoto City, Kyoto, 606-8502 Japan

Correction to: *Communications Biology* 10.1038/s42003-022-03917-5, published online 09 September 2022.

The original version of the Supplementary information associated with this Article was a marked-up version associated with the peer-review process of the Article. The Supplementary information file is now unmarked and corrected in the PDF and HTML versions of the Article.

## Supplementary information

Updated Supplementary Information

