# Supplementary material for: A RUNX-targeted gene switch-off approach modulates the BIRC5/PIF1-p21 pathway and reduces glioblastoma growth in mice
Source: Commun Biol. 2022 Sep 9;5:939. doi: 10.1038/s42003-022-03917-5 (PMC9463152; doi:10.1038/s42003-022-03917-5)
Supplement: Supplementary file 2 — Supplementary information [file 42003_2022_3917_MOESM2_ESM.pdf]

Supplementary Information

A RUNX-targeted gene switch-off approach modulates the BIRC5/PIF1-p21 pathway and reduces glioblastoma growth in mice

Etsuko Yamamoto Hattori[1,2], Tatsuya Masuda[2], Yohei Mineharu[1], Masamitsu Mikami[2,3], Yukinori Terada[1], Yasuzumi Matsui[1,2], Hirohito Kubota[3], Hidemasa Matsuo[2], Masahiro Hirata[4], Tatsuki R. Kataoka[4], Tatsutoshi Nakahata[5], Shuji Ikeda[6], Susumu Miyamoto[1], Hiroshi Sugiyama[6]*, Yoshiki Arakawa[1]*, Yasuhiko Kamikubo[2]*

[1] Department of Neurosurgery, Graduate School of Medicine, Kyoto University; Kyoto City, Kyoto 606-8507, Japan

[2] Department of Human Health Sciences, Graduate School of Medicine, Kyoto University; Kyoto City, Kyoto 606-8507, Japan

[3] Department of Pediatrics, Graduate School of Medicine, Kyoto University; Kyoto City, Kyoto 606-8507, Japan

[4] Department of Diagnostic Pathology, Kyoto University Hospital; Kyoto City, Kyoto 606-8507, Japan

[5] Drug Discovery Technology Development Office, Center for iPS Cell Research and Application (CiRA), Kyoto University; Kyoto City, Kyoto 606-8507, Japan

[6] Department of Chemistry, Graduate School of Science, Kyoto University; Kyoto City, Kyoto 606-8502, Japan

These authors contributed equally: Etsuko Yamamoto Hattori, Tatsuya Masuda.

*Corresponding authors

Hiroshi Sugiyama, E-mail: sugiyama.hiroshi.3s@kyoto-u.ac.jp

Yoshiki Arakawa, E-mail: yarakawa@kuhp.kyoto-u.ac.jp

Yasuhiko Kamikubo, E-mail: kamikubo.yasuhiko.7u@kuhp.kyoto-u.ac.jp

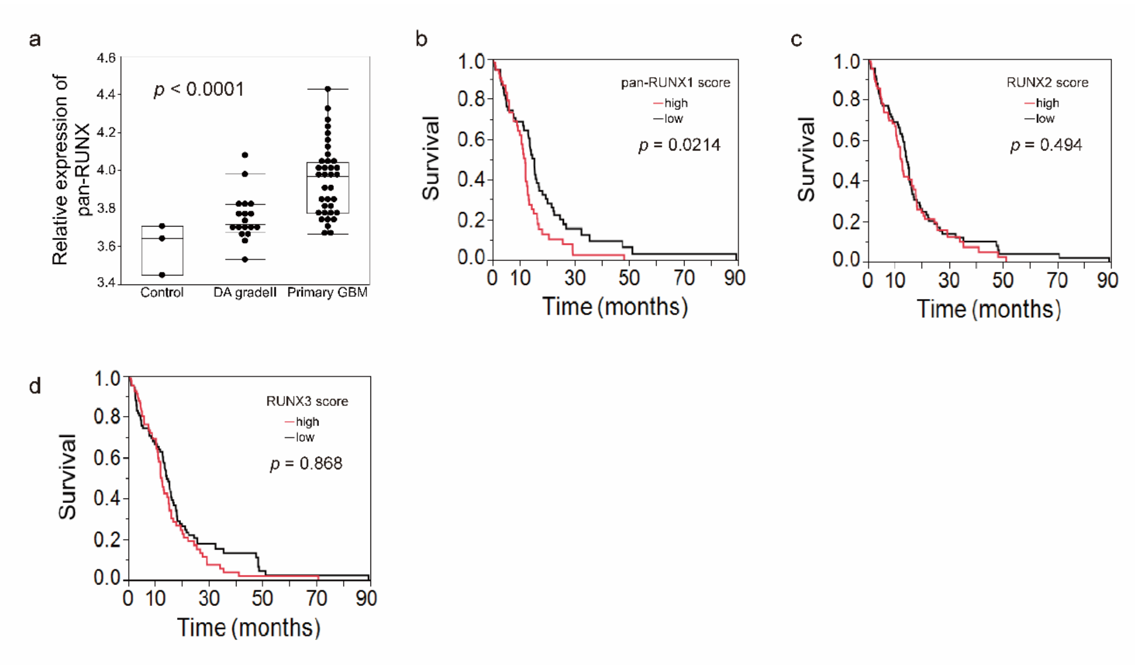

Supplementary Figure 1: Expression level and difference in survival rate of RUNX family.

a, Relative expression level of pan-RUNX to GAPDH for normal brain and each grade of glioma. Data were retrieved from GSE 111260. The box shows interquartile range. Upper border shows the upper quartile, middle line shows the median and lower border shows the lower quartile. Top and bottom lines show maximum and minimum values. *P* values were analysed by one-way ANOVA.

b-d, Survival curve based on glioblastoma pan-RUNX (**b**), RUNX2 (**c**) and RUNX3 (**d**) expression levels.

Numbers of subjects in the pan-RUNX high (top one-third) and low (bottom one-third) groups are 55 and 55, and numbers in RUNX2 and RUNX3 high (top half) and low

(bottom half) groups are 83 and 84, respectively. *P*-values were calculated by log-rank testing. Data were retrieved from The Cancer Genome Atlas (TCGA). The datasets are available in GDC TCGA Glioblastoma repository, https://gdc-hub.s3.us-east-1.amazonaws.com/latest/TCGA-GBM.htseq_fpkm.tsv.gz.

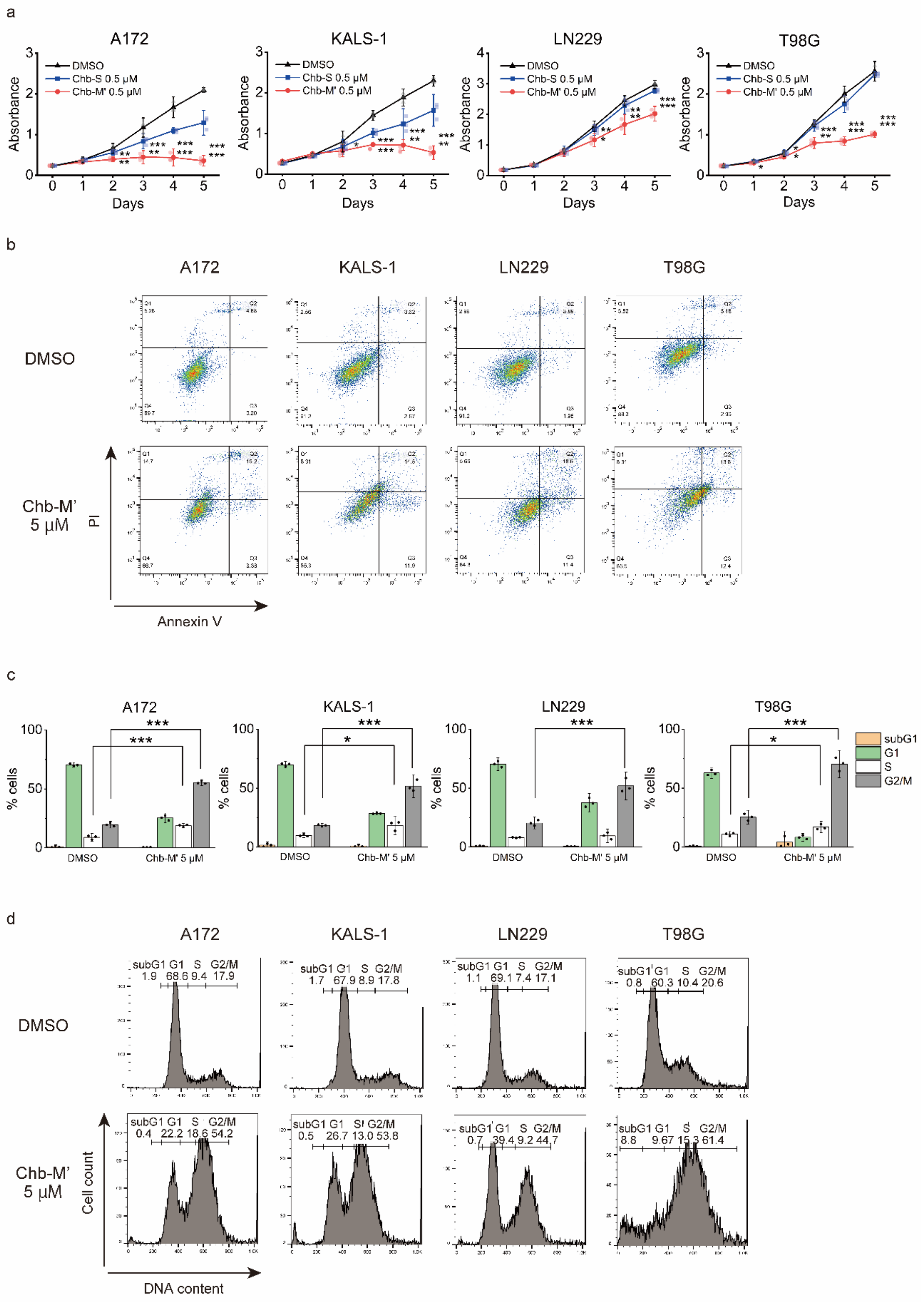
a
A172
KALS-1
LN229
T98G
DMSO
Chb-S 0.5 μM
Chb-M' 0.5 μM
Absorbance
Days
b
DMSO
Chb-M' 5 μM
PI
Annexin V
c
% cells
subG1
G1
S
G2/M
DMSO
Chb-M' 5 μM
d
Cell count
DNA content

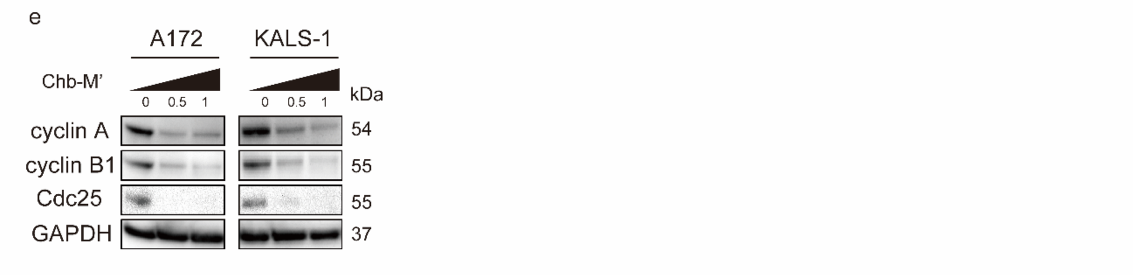

Supplementary Figure 2: Anti-tumour effects of Chb-M'.

a, Growth curves of GBM cells treated with Chb-M’ 0.5 μM and Chb-S 0.5 μM. N = 3.

b, Representative figures for apoptosis status as determined from A172, KALS-1, LN229 and T98G cell lines cultured in the presence of 5 μM of Chb-M' for 48 h.

c-d, Cell cycle arrest caused by Chb-M'. A172, KALS-1, LN229 and T98G cell lines were cultured as in **b**. **C** shows the percentage cell count for each cell cycle and **d** is the representative figure. n = 3.

e, Expression levels of genes associated with cell cycle arrest detected by immunoblotting. Cells were treated with 0.5 μM or 1 μM of Chb-M' or DMSO for 48 h, then cell lysates were prepared and immunoblotted.

Data represent mean ± 95%CI. *p < 0.05, **p < 0.01, ***p < 0.001, by two-tailed Student’s t-test.

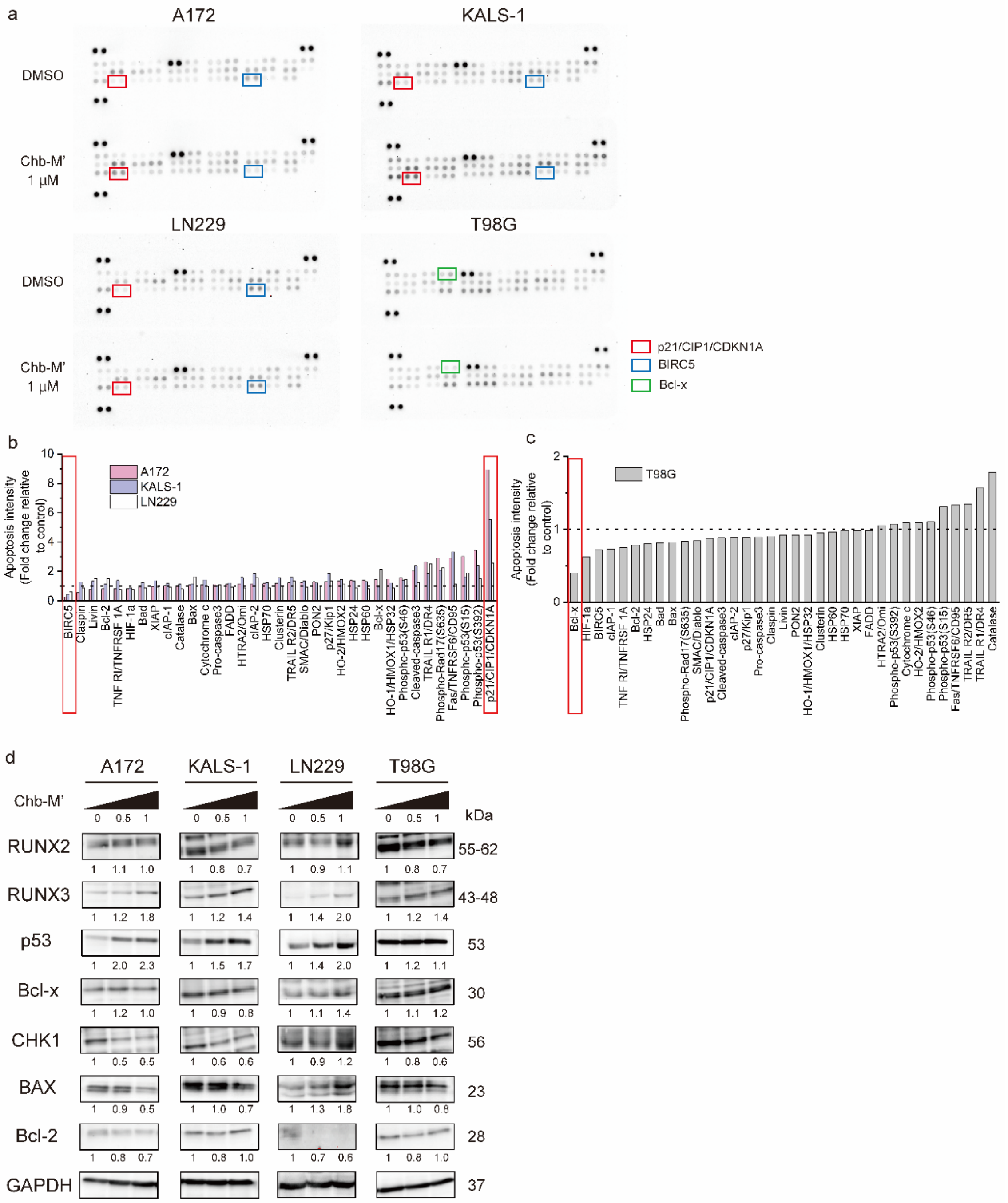
a
A172
KALS-1
DMSO
Chb-M'
1 μM
LN229
T98G
p21/CIP1/CDKN1A
BIRC5
Bcl-x
b
Apoptosis intensity
(Fold change relative
to control)
A172
KALS-1
LN229
BIRC5
Claspin
Livin
Bcl-2
TNF RI/TNFRSF 1A
HIF-1a
Bad
XIAP
cIAP-1
Catalase
Bax
Cytochrome c
Pro-caspase3
FADD
HTRA2/Omi
cIAP-2
HSP70
Clusterin
TRAIL R2/DR5
SMAC/Diablo
PON2
p27/Kip1
HO-2/HMOX2
HSP24
HSP60
Bcl-x
HO-1/HMOX1/HSP32
Phospho-p53(S46)
Cleaved-caspase3
TRAIL R1/DR4
Phospho-Rad17(S635)
Fas/TNFRSF6/CD95
Phospho-p53(S15)
Phospho-p53(S392)
p21/CIP1/CDKN1A
c
T98G
Bcl-x
HIF-1a
BIRC5
cIAP-1
TNF RI/TNFRSF 1A
Bcl-2
HSP24
Bad
Bax
Phospho-Rad17(S635)
SMAC/Diablo
p21/CIP1/CDKN1A
Cleaved-caspase3
cIAP-2
p27/Kip1
Pro-caspase3
Claspin
Livin
PON2
HO-1/HMOX1/HSP32
Clusterin
HSP60
HSP70
XIAP
FADD
HTRA2/Omi
Phospho-p53(S392)
Cytochrome c
HO-2/HMOX2
Phospho-p53(S46)
Phospho-p53(S15)
Fas/TNFRSF6/CD95
TRAIL R2/DR5
TRAIL R1/DR4
Catalase
d
A172
KALS-1
LN229
T98G
Chb-M'
0 0.5 1
kDa
RUNX2
55-62
RUNX3
43-48
p53
53
Bcl-x
30
CHK1
56
BAX
23
Bcl-2
28
GAPDH
37

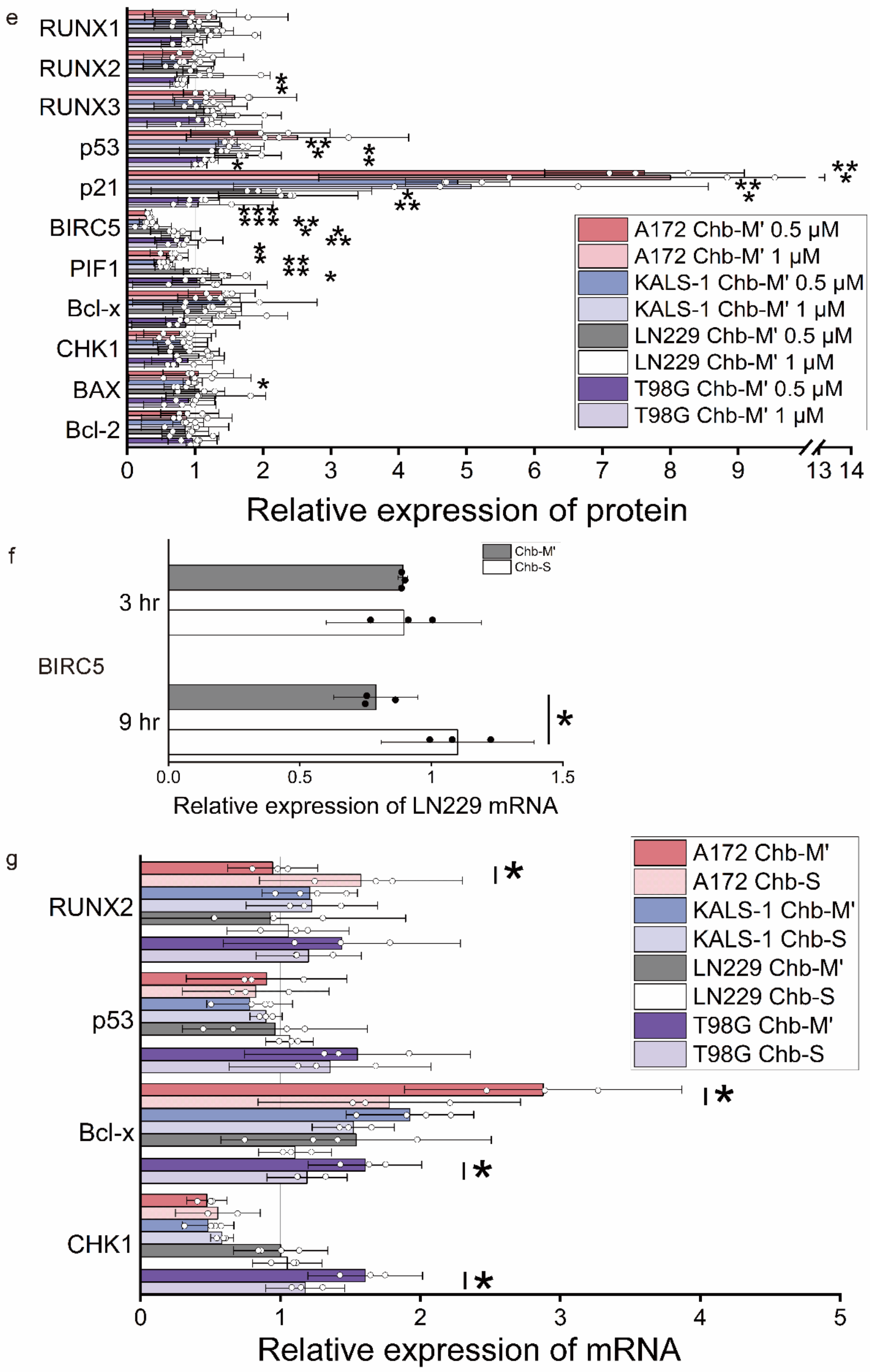
e
RUNX1
RUNX2
RUNX3
p53
p21
BIRC5
PIF1
Bcl-x
CHK1
BAX
Bcl-2
A172 Chb-M' 0.5 μM
A172 Chb-M' 1 μM
KALS-1 Chb-M' 0.5 μM
KALS-1 Chb-M' 1 μM
LN229 Chb-M' 0.5 μM
LN229 Chb-M' 1 μM
T98G Chb-M' 0.5 μM
T98G Chb-M' 1 μM
0 1 2 3 4 5 6 7 8 9 13 14
Relative expression of protein
f
Chb-M'
Chb-S
3 hr
BIRC5
9 hr
0.0 0.5 1 1.5
Relative expression of LN229 mRNA
g
RUNX2
p53
Bcl-x
CHK1
A172 Chb-M'
A172 Chb-S
KALS-1 Chb-M'
KALS-1 Chb-S
LN229 Chb-M'
LN229 Chb-S
T98G Chb-M'
T98G Chb-S
0 1 2 3 4 5
Relative expression of mRNA

Supplementary Figure 3: Selection of genes that fluctuate in relation to Chb-M'.

a-c, Relative densitometric quantification of apoptosis array spots in Chb-M'-treated GBM cells compared to the control (**b**: A172, KALS-1 and LN229; **c**: T98G). Cells were treated with DMSO or 1 μM Chb-M' for 48 h, then lysed for the apoptosis array. Each receptor was spotted in duplicate. **A** shows the immunoblot image. Blue, red and green squares indicate spots of BIRC5, p21 and Bcl-x, respectively.

d, Immunoblotting of each GBM cell line treated with 0.5 μM or 1 μM Chb-M' or DMSO for 48 h. The numbers under the blot are normalized values for GAPDH and DMSO-treated cells.

e, Protein expression levels of the immunoblotting were quantitatively measured and corrected with GAPDH. Values are normalized to DMSO-treated cells. Cells were treated as in **d**. n = 3.

f, Gene expression levels of real-time RT-PCR. LN229 cells were treated with 1 μM of Chb-M' 1 μM of Chb-S or DMSO for 3, 9 h, then total RNA was prepared. Values of Chb-M' and Chb-S are normalized to DMSO-treated cells. n = 3.

g, Gene expression levels of real-time RT-PCR. Cells were treated with 1 μM of Chb-M' or 1 μM of Chb-S or DMSO for 48 h, then total RNA was prepared. Values of Chb-M' and Chb-S are normalized to DMSO-treated cells. KALS-1 treated Chb-M': N = 4,

other samples: $n = 3$.

Data represent mean ± 95% confidence interval (CI). *$p < 0.05$, **$p < 0.01$, ***$p < 0.001$, by Welch's t-test (**e**), two-tailed Student's t-test (**f, g**).

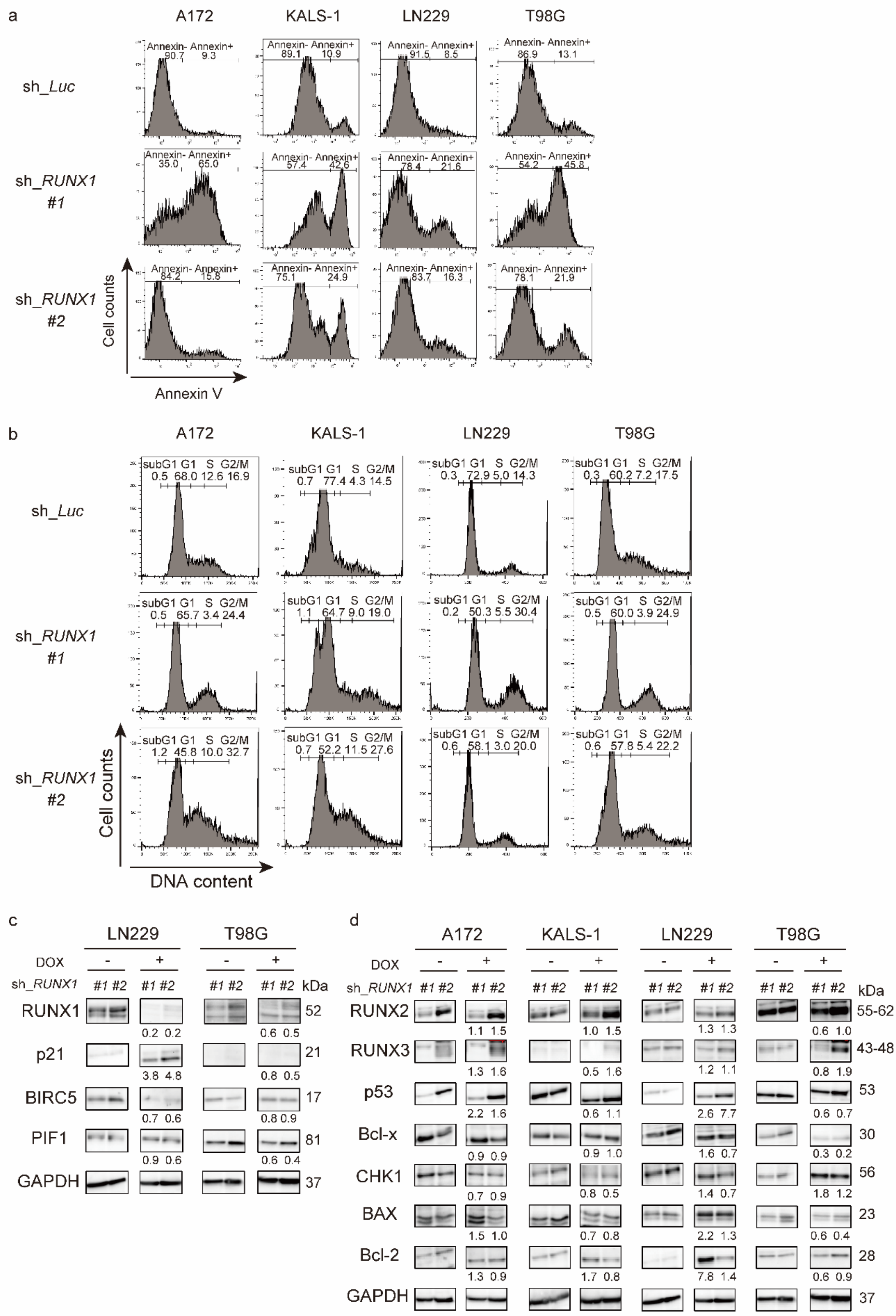
a
A172
KALS-1
LN229
T98G
sh_Luc
sh_RUNX1 #1
sh_RUNX1 #2
Annexin- Annexin+
90.7 9.3
89.1 10.9
91.5 8.5
86.9 13.1
35.0 65.0
57.4 42.6
78.4 21.6
54.2 45.8
84.2 15.8
75.1 24.9
83.7 16.3
78.1 21.9
Cell counts
Annexin V
b
A172
KALS-1
LN229
T98G
sh_Luc
sh_RUNX1 #1
sh_RUNX1 #2
subG1 G1 S G2/M
0.5 68.0 12.6 16.9
0.7 77.4 4.3 14.5
0.3 72.9 5.0 14.3
0.3 60.2 7.2 17.5
0.5 65.7 3.4 24.4
1.1 64.7 9.0 19.0
0.2 50.3 5.5 30.4
0.5 60.0 3.9 24.9
1.2 45.8 10.0 32.7
0.7 52.2 11.5 27.6
0.6 58.1 3.0 20.0
0.6 57.8 5.4 22.2
Cell counts
DNA content
c
LN229
T98G
DOX - + - +
sh_RUNX1 #1 #2 #1 #2 #1 #2 #1 #2
kDa
RUNX1 52
0.2 0.2 0.6 0.5
p21 21
3.8 4.8 0.8 0.5
BIRC5 17
0.7 0.6 0.8 0.9
PIF1 81
0.9 0.6 0.6 0.4
GAPDH 37
d
A172
KALS-1
LN229
T98G
DOX - + - + - + - +
sh_RUNX1 #1 #2 #1 #2 #1 #2 #1 #2 #1 #2 #1 #2 #1 #2 #1 #2
kDa
RUNX2 55-62
1.1 1.5 1.0 1.5 1.3 1.3 0.6 1.0
RUNX3 43-48
1.3 1.6 0.5 1.6 1.2 1.1 0.8 1.9
p53 53
2.2 1.6 0.6 1.1 2.6 7.7 0.6 0.7
Bcl-x 30
0.9 0.9 0.9 1.0 1.6 0.7 0.3 0.2
CHK1 56
0.7 0.9 0.8 0.5 1.4 0.7 1.8 1.2
BAX 23
1.5 1.0 0.7 0.8 2.2 1.3 0.6 0.4
Bcl-2 28
1.3 0.9 1.7 0.8 7.8 1.4 0.6 0.9
GAPDH 37

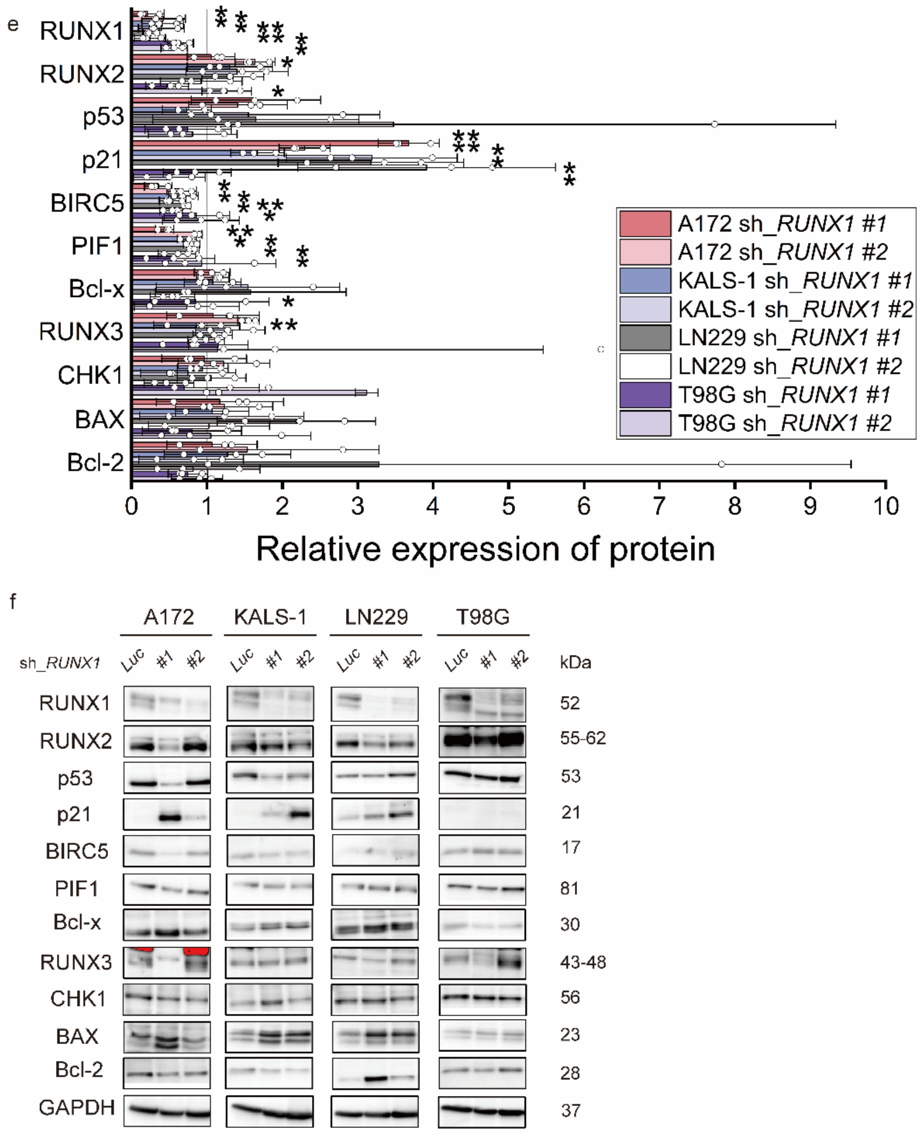

Supplementary Figure 4: Apoptosis and cell cycle arrest are induced by decreased expression of RUNX1.

a, Representative figures of apoptosis status determined in four glioblastoma cell lines transduced with control or *RUNX1* shRNAs. Cells were treated with 5 μM of

doxycycline for 4 days (LN229 and T98G) or 6 days (A172 and LN229).

b, Representative figures of cell cycle assay determined in four glioblastoma cell lines transduced with control or *RUNX1* shRNAs. All cells were treated with 5 μM of doxycycline for 4 days.

c-d, Immunoblotting of each GBM cell line transduced with *RUNX1* shRNAs. Sh_*RUNX1* cells were cultured in the presence of 5 μM of doxycycline and control cells were cultured in the absence of doxycycline. The numbers under the blot are normalized values for GAPDH and control cells.

e, Protein expression levels of the immunoblotting were quantitatively measured and corrected with GAPDH. Values are normalized to doxycycline-untreated cells. Cells were treated as in **c** and **d**. n = 3.

f, Immunoblotting of each GBM cell line transduced with control or *RUNX1* shRNAs. Cells were cultured in the presence of 5 μM of doxycycline.

Data represent mean ± 95%CI. *p < 0.05, **p < 0.01, by Welch's t-test.

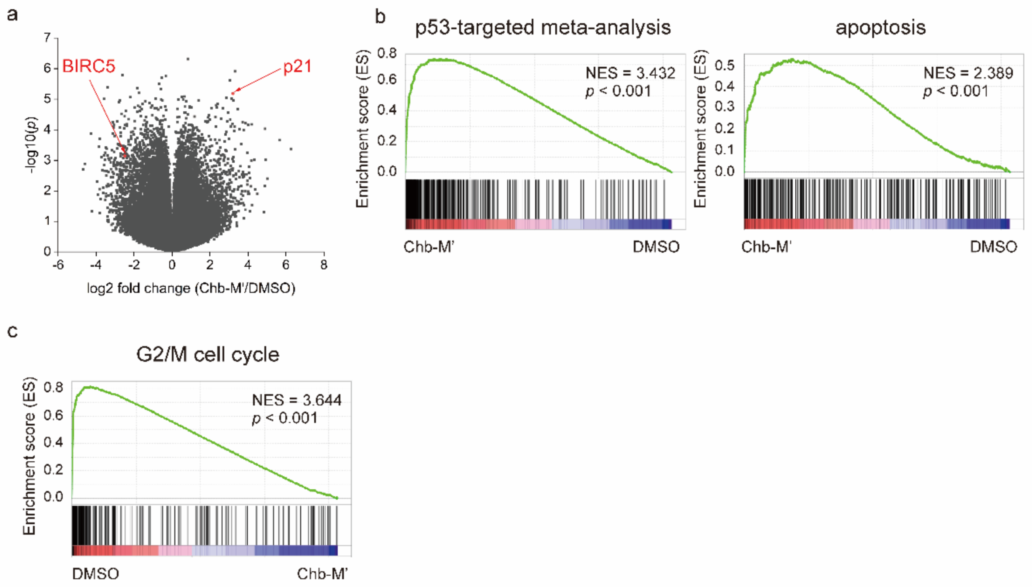

Supplementary Figure 5: Genes involved in apoptosis and cell cycle arrest are altered by Chb-M'.

a, Microarray results for 1 µM of Chb-M' against DMSO are shown in a volcano plot. BIRC5 and p21 are indicated by red dots. n = 3.

b-c, Microarray results were subsequently analysed by GSEA. **B** shows Chb-M'-treated samples were enriched in p53 and apoptosis-related genes. **C** shows Chb-M'-treated samples depleted for G2/M cell cycle-related genes.

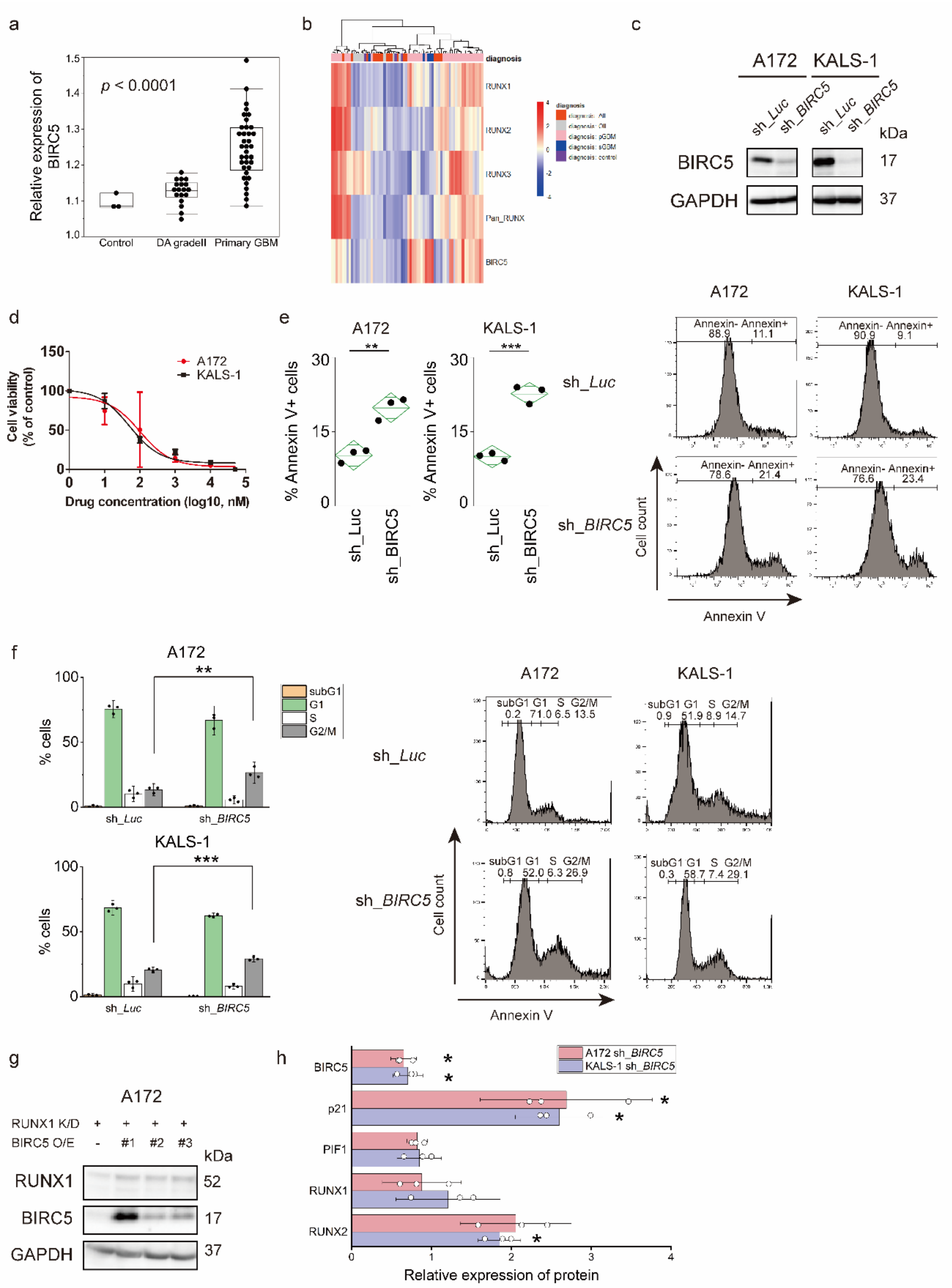

Supplementary Figure 6: Apoptosis and cell cycle arrest are induced by decreased expression of BIRC5

a, Relative expression levels of BIRC5 normalized to GAPDH for normal brain and each grade of glioma. Data were retrieved from GSE 111260. The box shows interquartile range. Upper border shows the upper quartile, middle line shows the median and lower border shows the lower quartile. Top and bottom lines indicate maximum and minimum values.

b, Clustering analysis to assess correlations between the RUNX family and BIRC5. The upper belt shows the grade of glioma. Data were retrieved from GSE 111260 microarray datasets.

c, Immunoblotting of each GBM cell line transduced with control or *BIRC5* shRNAs. Control (sh_*Luc*) and sh_*BIRC5* cells were cultured in the presence of 5 μM of doxycycline.

d, Dose-response curves of A172 and KALS-1 cell lines after treatment with YM155 at 72 h. IC_{50} values of YM155 are shown in table S3. IC_{50} values were fit to data calculated using GraphPad Prism 5 software. n = 3.

e, BIRC5 depression induces apoptosis. Non-depressed and *BIRC5*-depressed A172 and KALS-1 cell lines were treated in the presence of 5 μM of doxycycline for 4 days. Left shows the percentage of Annexin V+ cells. Right shows a representative figure of apoptosis status. The line in the centre of the rhombus indicates the mean value, and the

vertical width indicates the 95%CI. n = 3.

f, BIRC5 depression induces G2/M arrest. Left shows the percentage of cells in each cell cycle. Right shows a representative figure. Non-depressed and *BIRC5*-depressed A172 and KALS-1 cells were treated as in **e**. n = 3.

g, Immunoblotting of *RUNX1*-depleted A172 cells with restored BIRC5 expression. The indicated cells were cultured in the presence of 5 μM of doxycycline.

h, Protein expression levels of the immunoblotting were quantitatively measured and corrected with GAPDH. Values are normalized to doxycycline-untreated cells. Non-depressed and *BIRC5*-depressed cells were cultured in the presence of 5 μM of doxycycline for 5 days. n = 3.

Data represent mean ± 95%CI. $^{**}p < 0.01$, $^{***}p < 0.001$, by one-way ANOVA (**a**), two-tailed Student's t-test (**e** and **f**), Welch's t-test. (h).

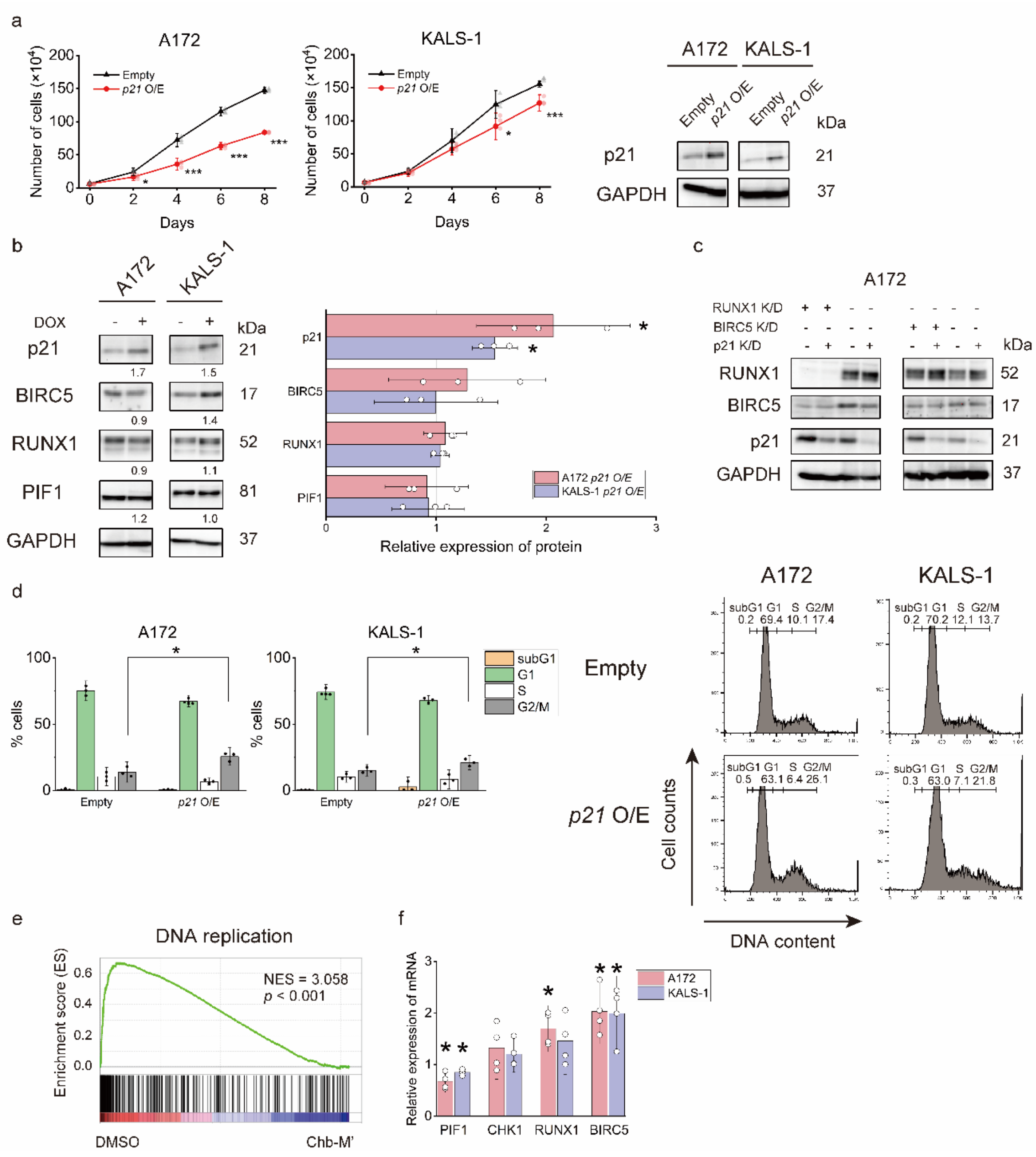

Supplementary Figure 7: Apoptosis and cell cycle arrest are induced by overexpression of p21 or suppressed PIF1.

a, Growth curves (left) and immunoblotting (right) of A172 and KALS-1 cell lines transduced with control (Empty vector) or with *p21* overexpression in the presence of 5

μM of doxycycline. n = 3.

b, Immunoblotting (left) and relative protein expression levels (right) of *p21* overexpression cells. Expressions of the RUNX family, BIRC5 and PIF1 were unaffected by p21 overexpression. Non-depressed and *BIRC5*-depressed cells were cultured in the presence of 5 μM of doxycycline for 4 days. The numbers under the blot are normalized values for GAPDH and cells cultured without doxycycline. Protein expression levels were quantitatively measured and corrected with GAPDH. Values are normalized to doxycycline-untreated cells.

c, Immunoblotting for *RUNX1*- or *BIRC5*-depleted A172 cells suppressed p21 expression. The indicated cells were cultured in the presence of 5 μM of doxycycline.

d, Overexpression of p21 induced G2/M arrest. Non-overexpressing and *p21*-overexpressing A172 and KALS-1 lines were treated in the presence of 5 μM of doxycycline for 6 days. Left shows percentages of cells in each cell cycle. Right shows a representative figure. n = 3.

e, Microarray results were subsequently analysed by GSEA. Chb-M'-treated samples depleted for DNA replication-related genes.

f, Expression levels of genes when PIF1 was repressed as detected by RT-PCR. PIF1 inhibition did not change CHK1 expression levels in A172 or KALS-1 cell lines. Cells

were cultured in the presence of *PIF1* siRNA for 3 days. n = 4.

Data represent mean ± 95%CI. *p < 0.05, **p < 0.01, ***p < 0.001, by two-tailed Student's t-test (**a**, **d**), Welch's t-test (**b, f**).

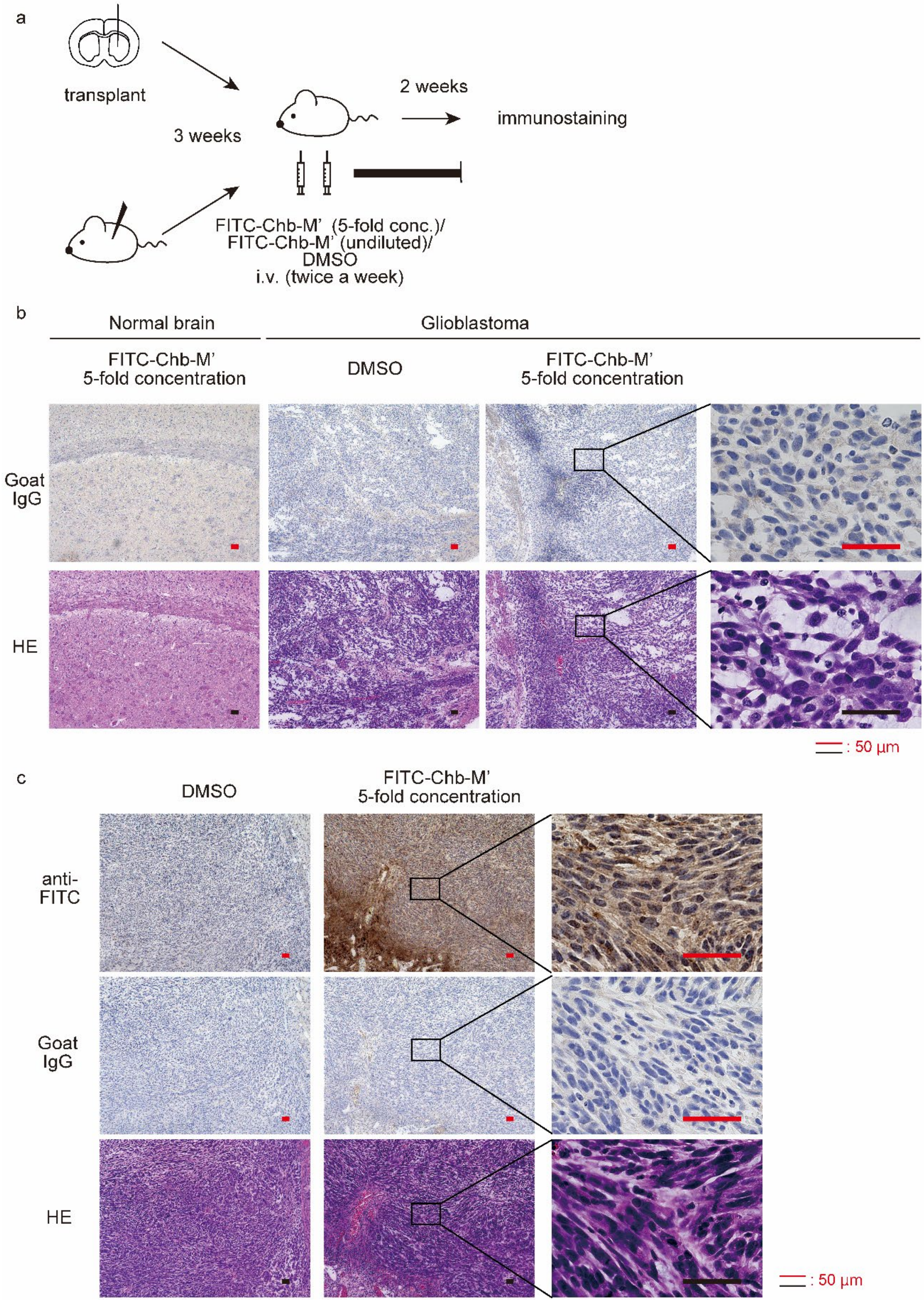
a
transplant
3 weeks
2 weeks
immunostaining
FITC-Chb-M' (5-fold conc.)/
FITC-Chb-M' (undiluted)/
DMSO
i.v. (twice a week)
b
Normal brain
Glioblastoma
FITC-Chb-M'
5-fold concentration
DMSO
FITC-Chb-M'
5-fold concentration
Goat
IgG
HE
: 50 μm
c
DMSO
FITC-Chb-M'
5-fold concentration
anti-
FITC
Goat
IgG
HE
: 50 μm

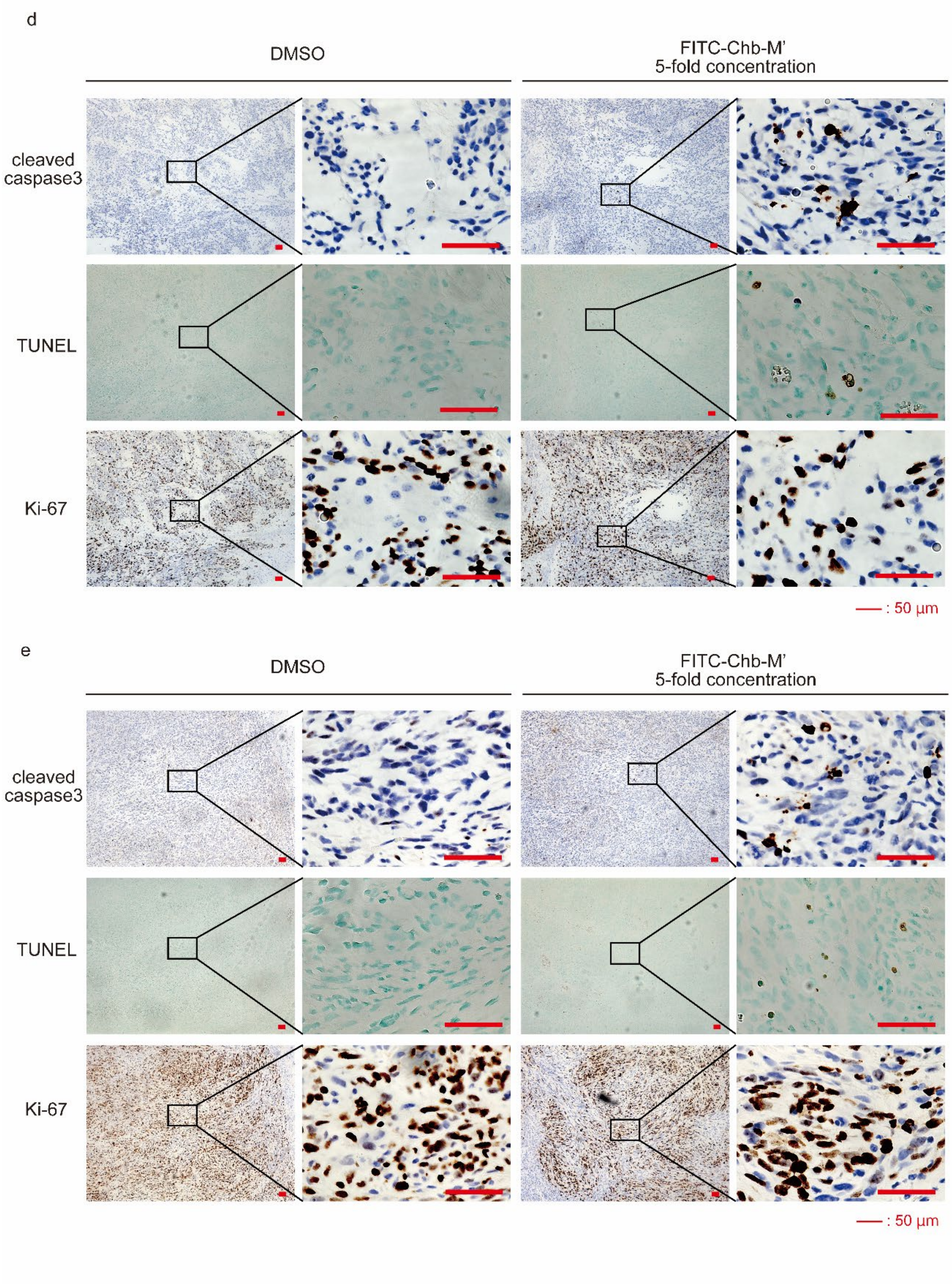

Supplementary Figure 8: Chb-M' is effective for intracranial tumour in vivo.

a, Schematic representation of treatment schedule in xenotransplanted mice.

b, Pathological samples of normal brain and intracranial tumour treated with FITC-labelled Chb-M' and DMSO immunostained with isotype-matched control antibody. Scale bars: 50 μm.

c, Pathological samples of subcutaneous tumour treated with FITC-labelled Chb-M' and DMSO immunostained with goat anti-FITC antibody (upper) and isotype-matched control antibody (middle). Scale bars: 50 μm. HE; Hematoxylin Eosin

d,e, Pathological samples of intracranial tumour(**d**) and subcutaneous tumour(**e**) treated with FITC-labelled Chb-M' and DMSO immunostained with cleaved caspase3 antibody (upper), TUNEL assay (middle) and Ki-67 antibody (lower). Scale bars: 50 μm.

Supplementary Figure 9 (Unedited Gels)

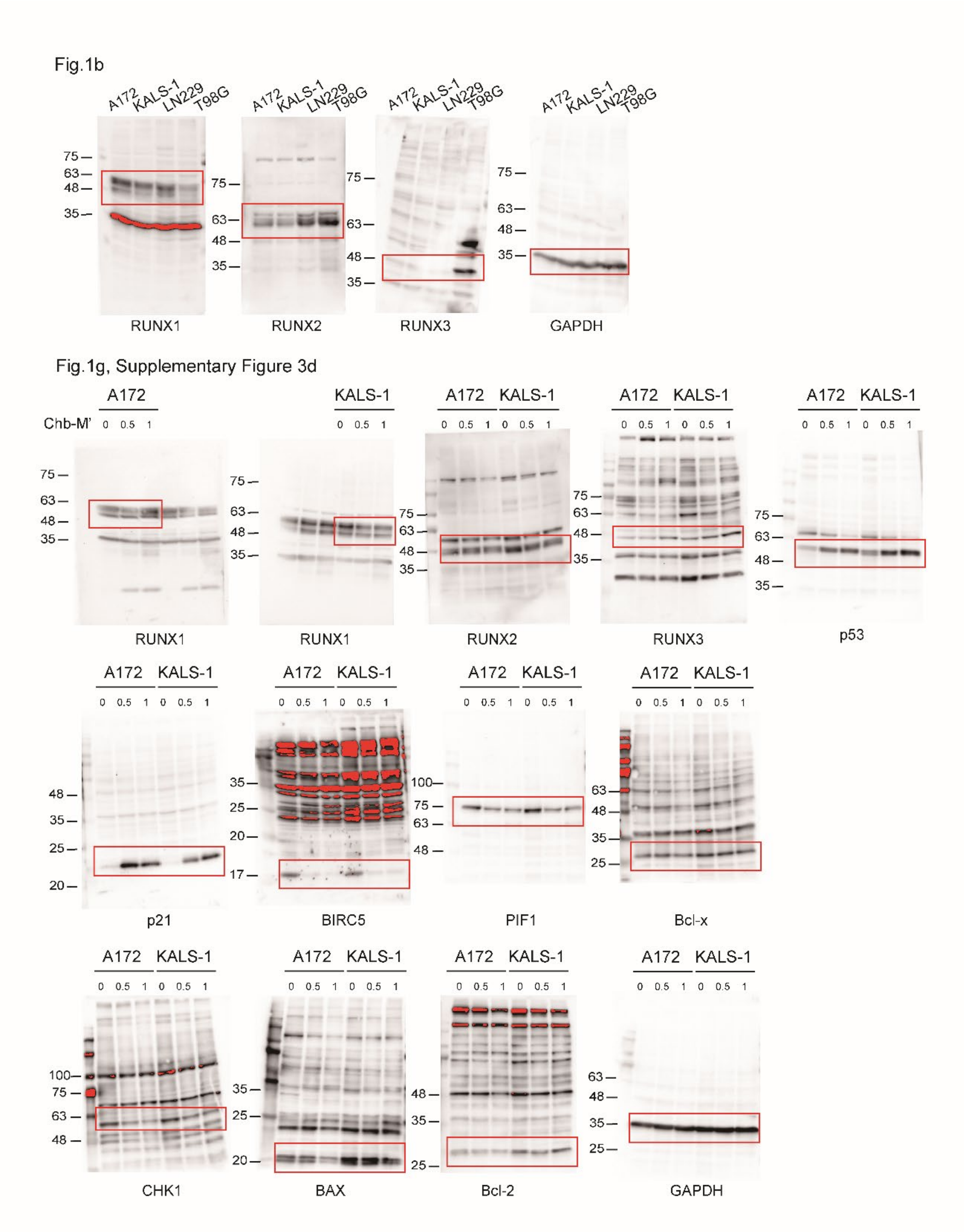

Fig.1g, Supplementary Figure 3d

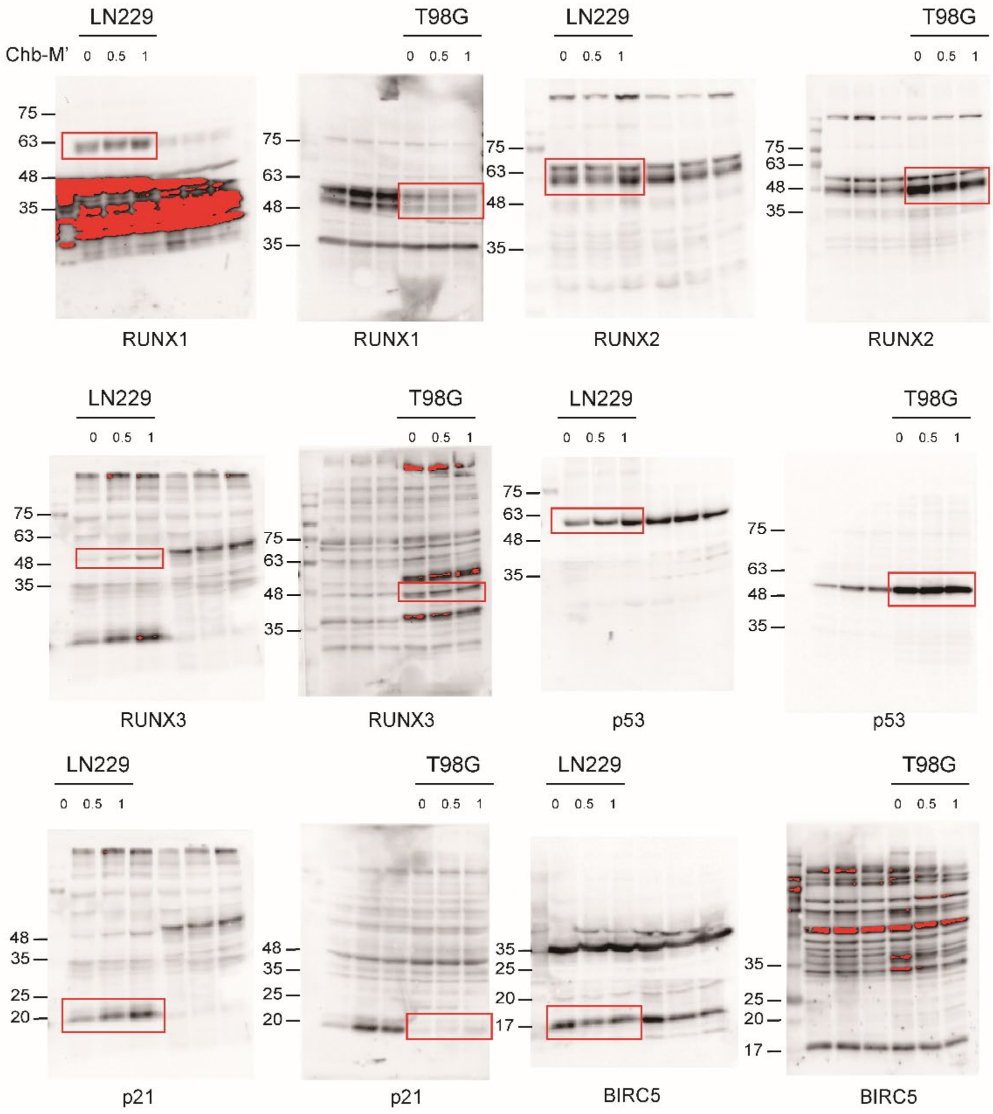

Fig.1g, Supplementary Figure 3d

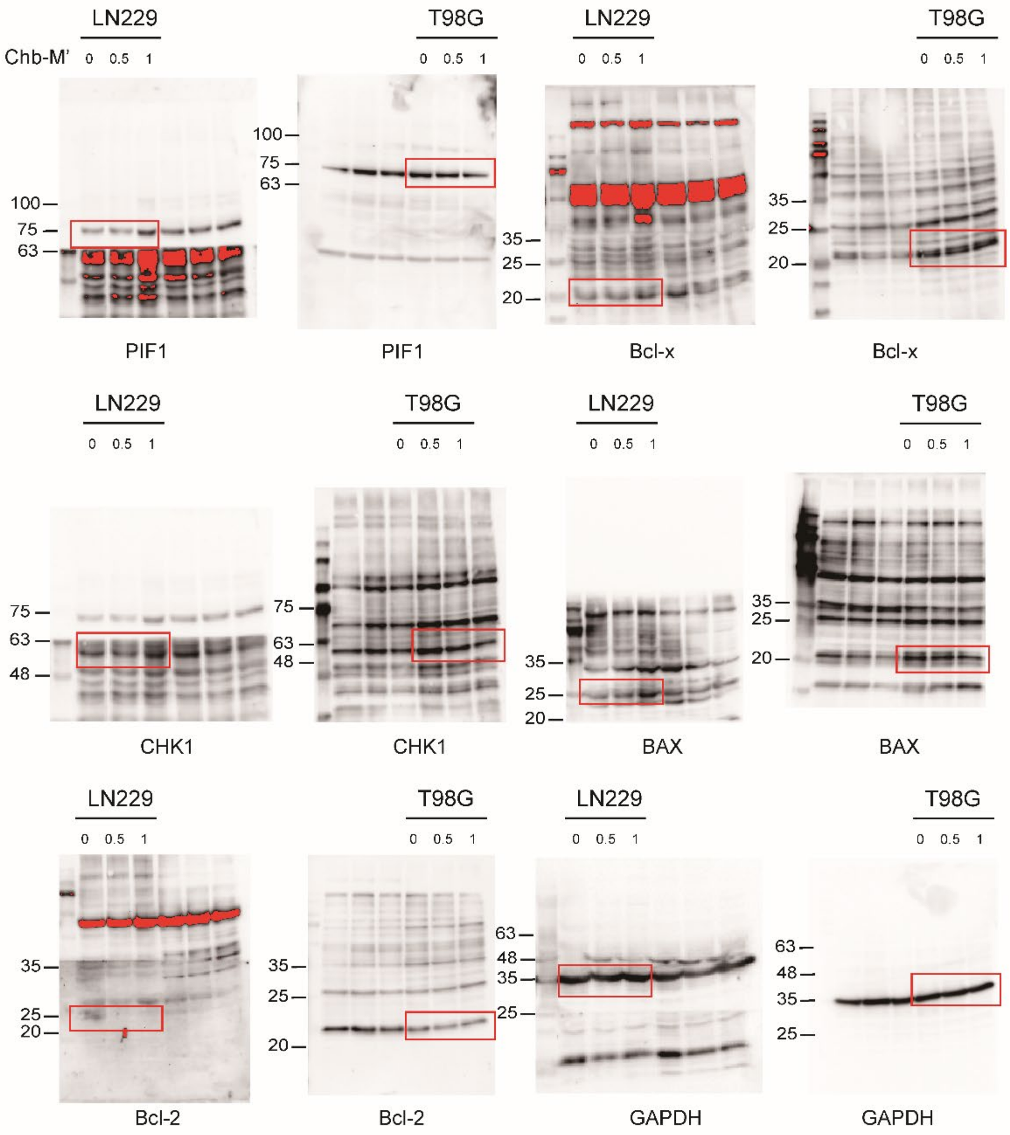

Supplementary Figure 2e

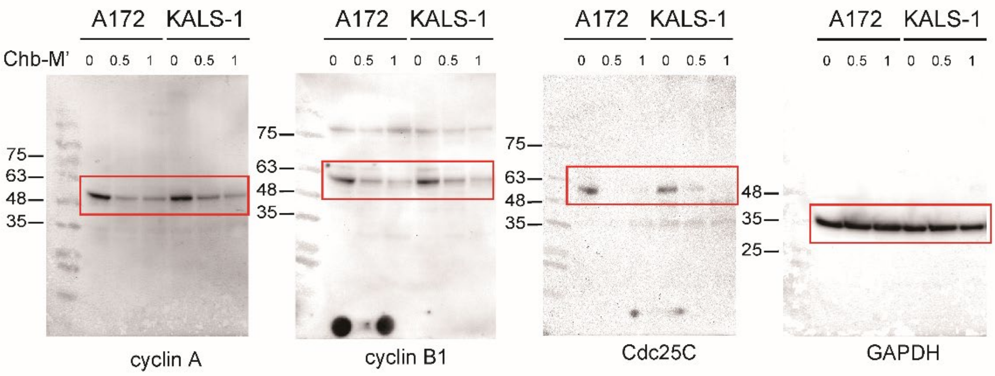

Fig. 2a, Fig. 3a, Supplementary Figure 4c,d,f

A172

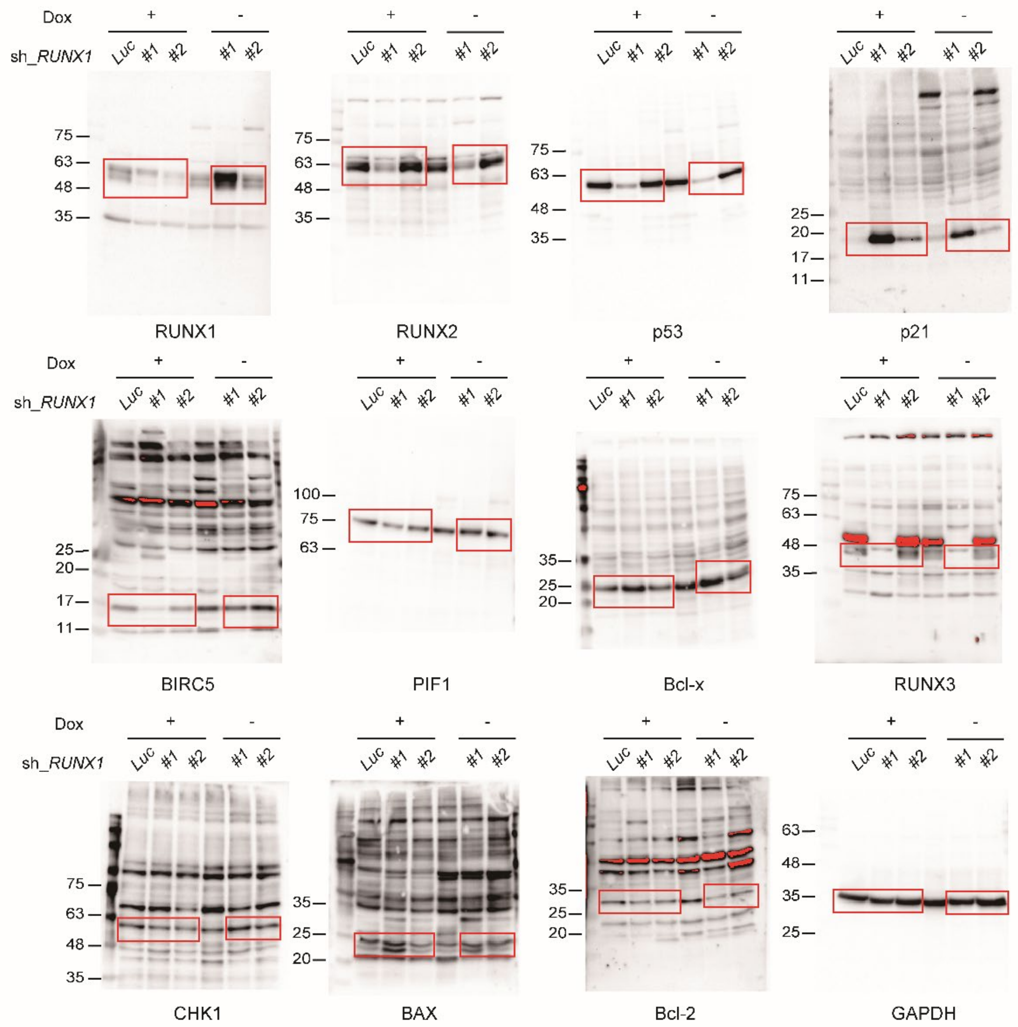

Fig. 2a, Fig. 3a, Supplementary Figure 4c,d

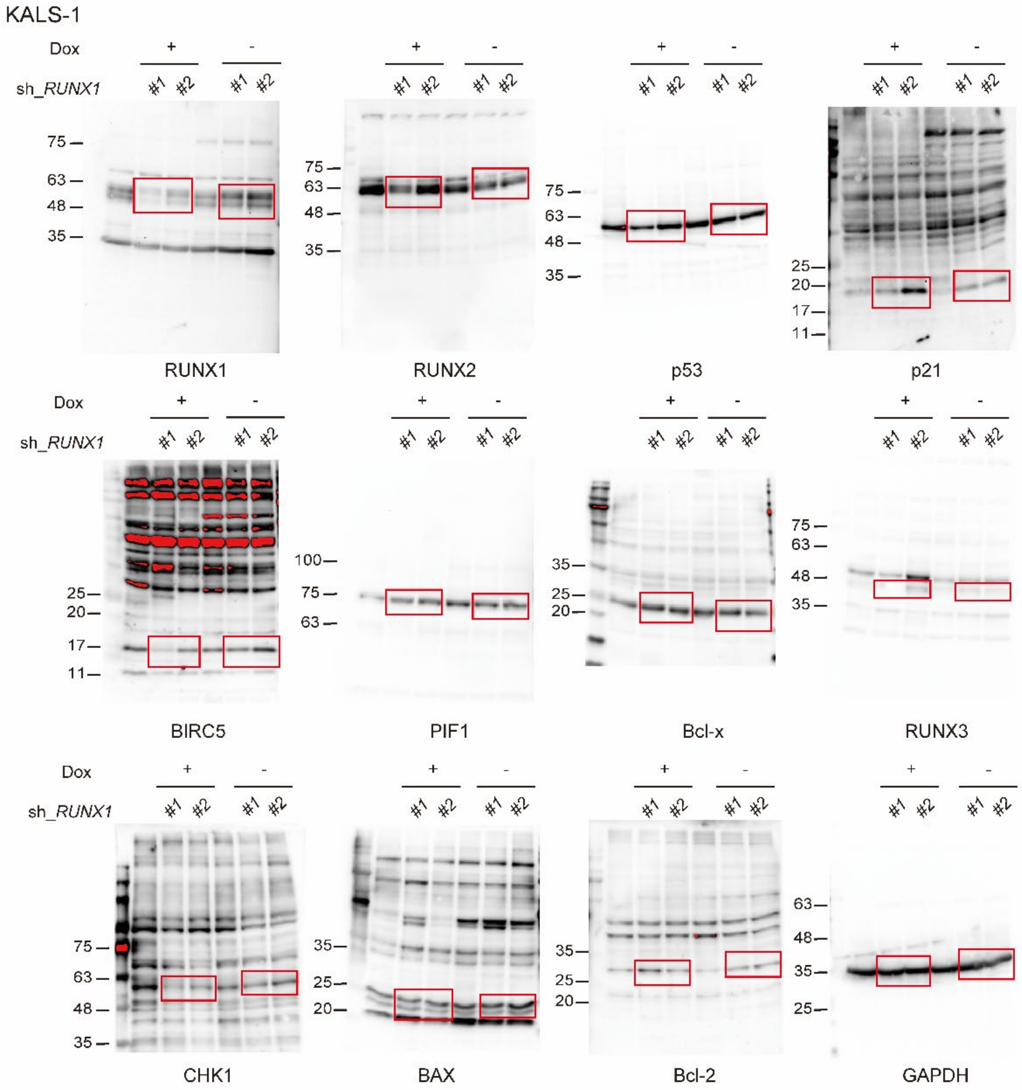

Supplementary Figure 4f

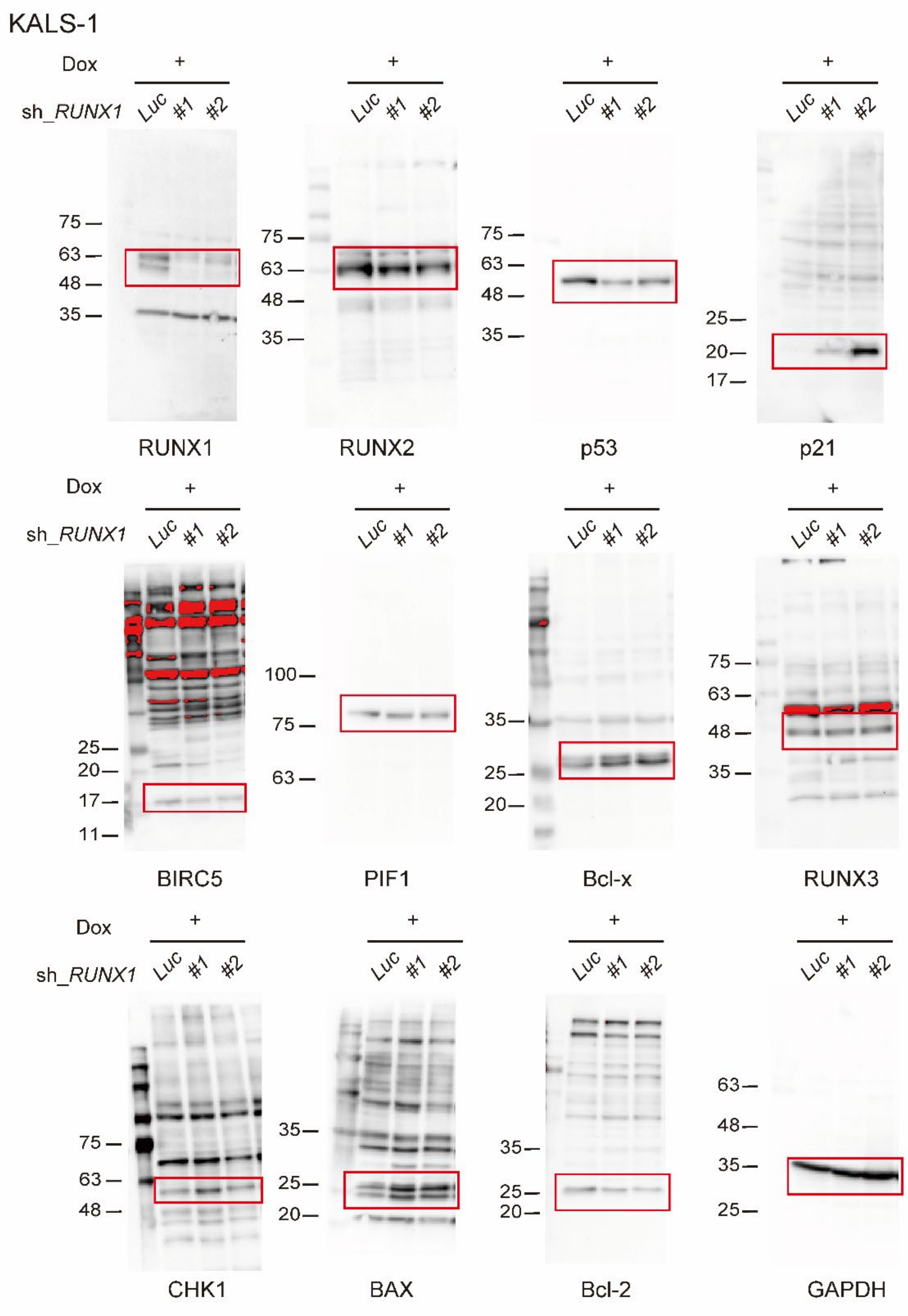

Fig. 2a, Supplementary Figure 4 c,d,f

LN229

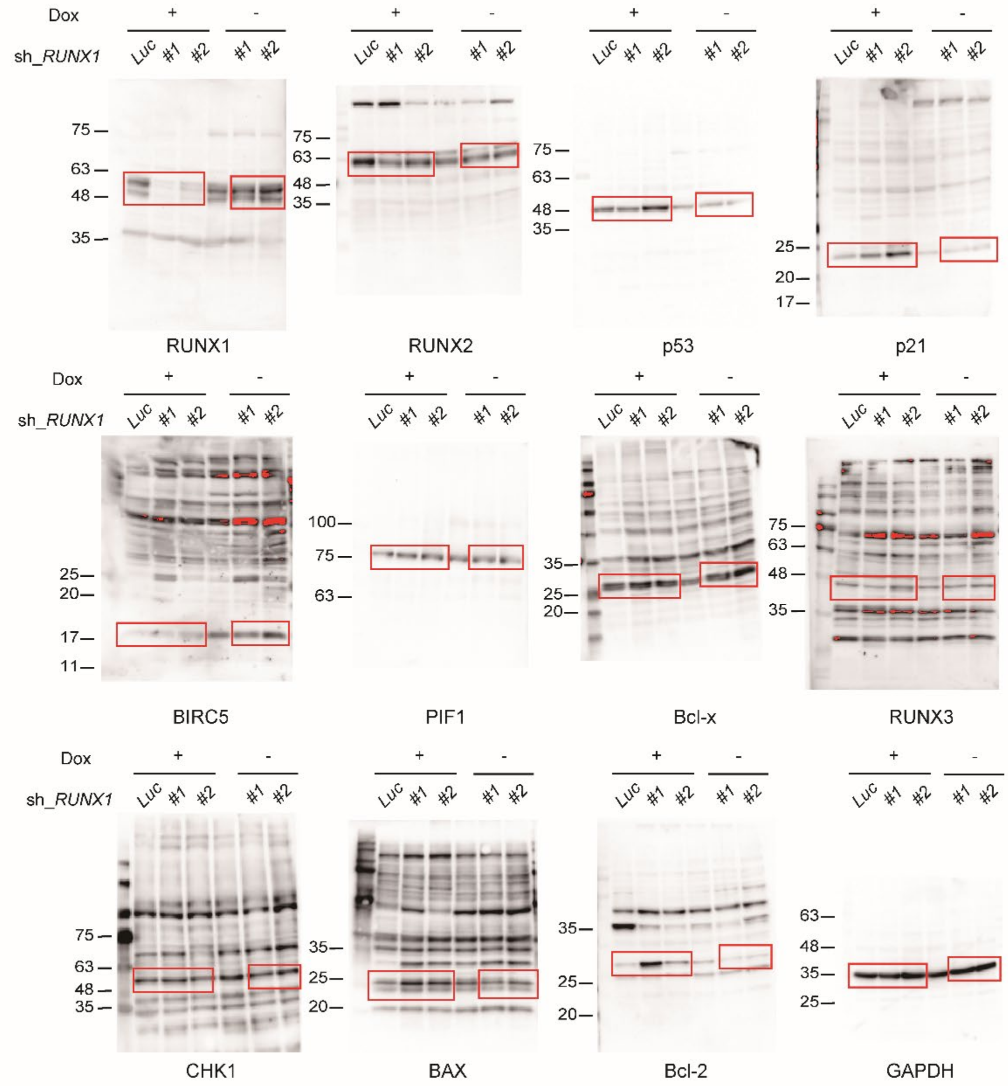

Fig. 2a, Supplementary Figure 4 c,d,f

T98G

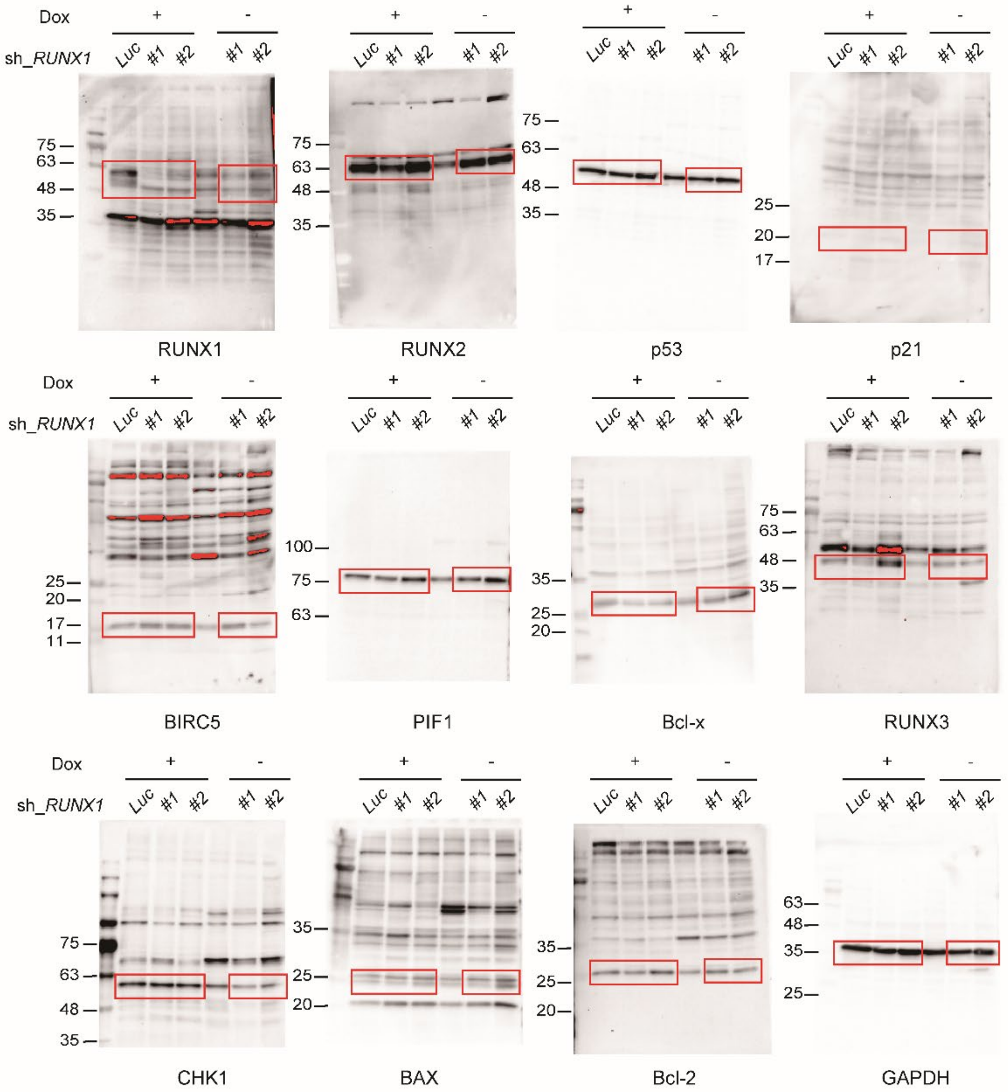

Supplementary Figure 6 c

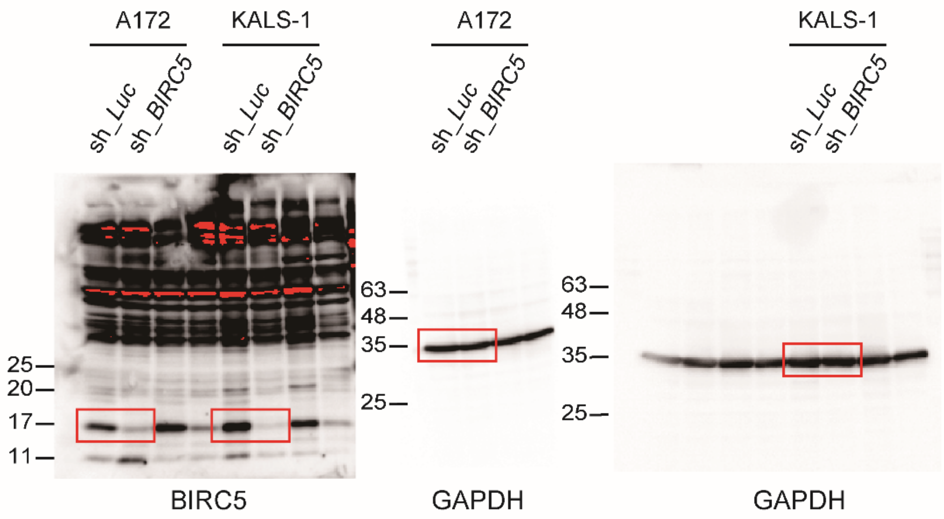

Supplementary Figure 6 g

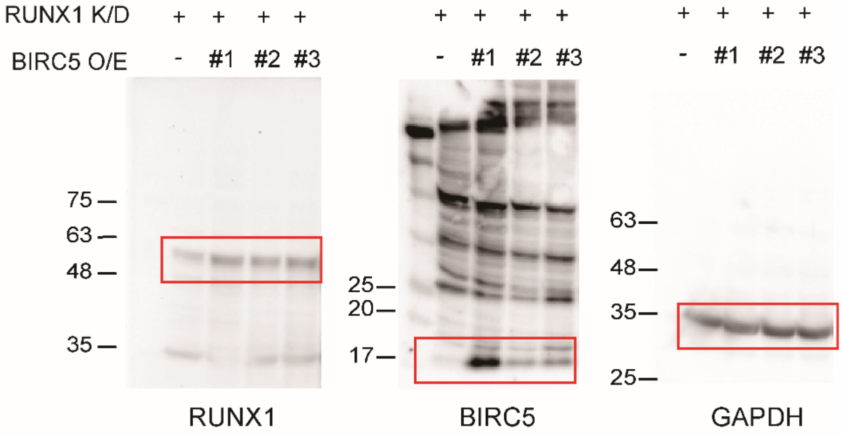

Fig. 3g

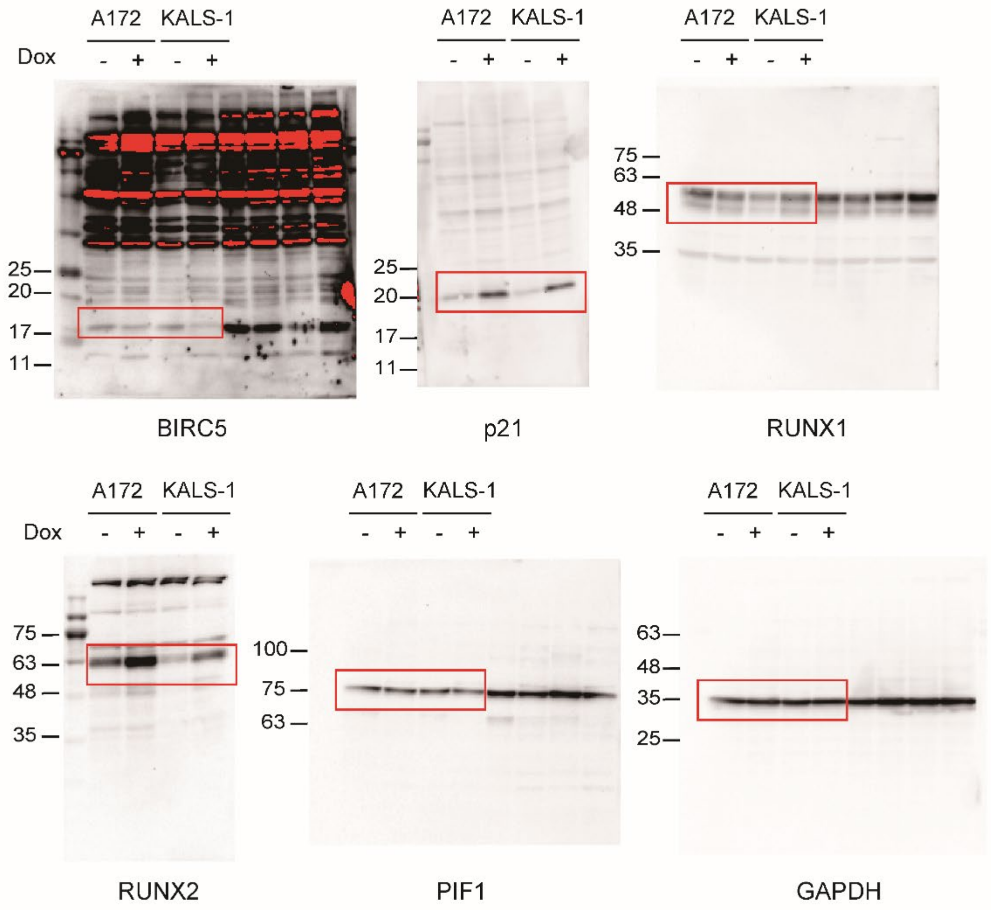

Supplementary Figure 7a

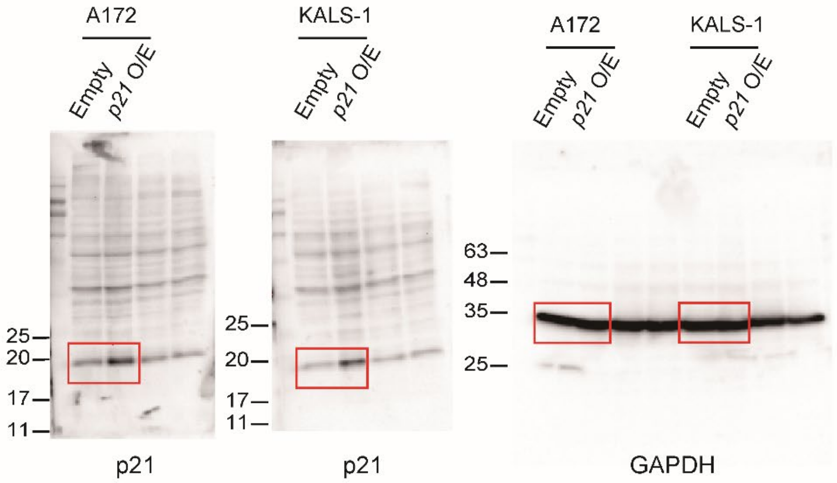

Supplementary Figure 7b

A172

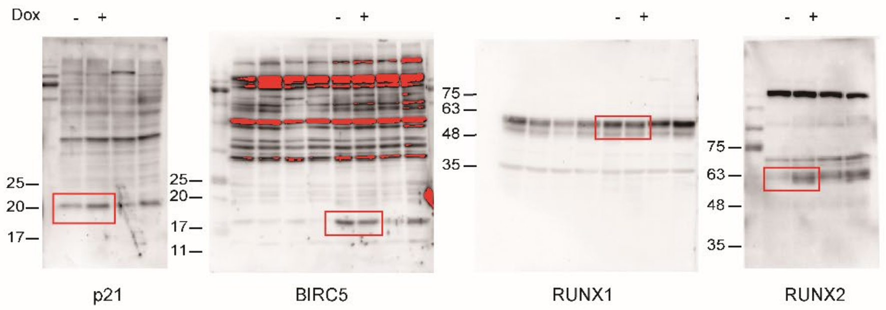

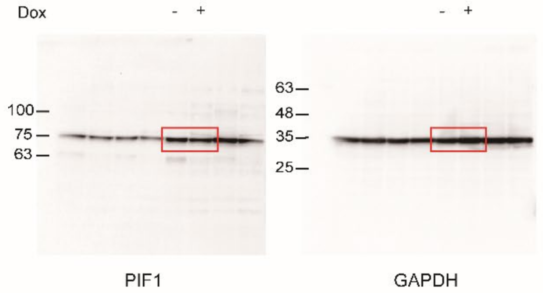

KALS-1

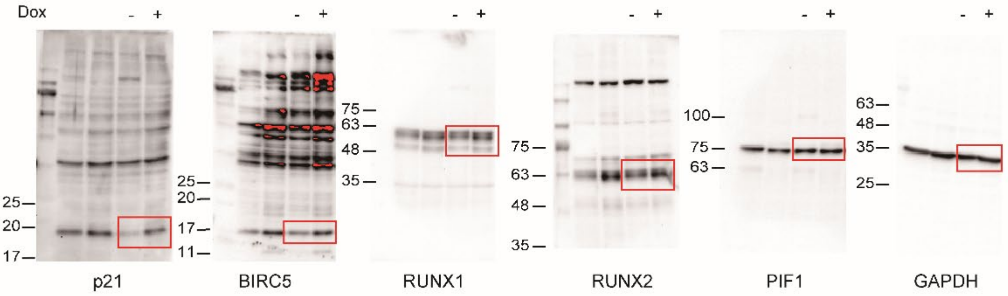

Supplementary Figure 7c

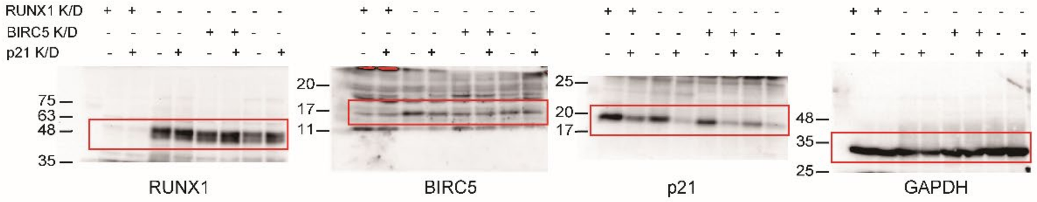

Supplementary Table 1. TP53 mutations in GBM cell lines

Cell line	Exon 4	Exon 6	Exon 7	Exon 10	Exon 11
A172	c.215 C>G<br>p.P72R<br>COSM3766190	-	-	-	-
KALS-1	-	-	c.722C>T<br>p.S241F<br>COSM10812	-	-
LN229	c.293 C>T<br>p.P98L<br>COSM44681	-	-	-	-
T98G	-	-	c.711G>A<br>p.M237I<br>COSM10834	-	-

Supplementary Table 2. Half maximal inhibitory concentration (IC_{50}) values for chlorambucil (Chb), Chb-M' and Chb-S and chlorambucil (Chb)

A172

IC_{50} (μM)	48 h	72 h	96 h
Chb	29.35	> 50	> 50
Chb-M'	25.71	2.47	0.85
Chb-S	25.73	17.75	2.51

KALS-1

IC_{50} (μM)	48 h	72 h	96 h
Chb	> 50	> 50	> 50
Chb-M'	14.88	1.49	0.15
Chb-S	26.67	2.48	2.63

LN229

IC_{50} (μM)	48 h	72 h	96 h
Chb	29.35	> 50	> 50
Chb-M'	11.13	3.35	1.39
Chb-S	> 50	> 50	> 50

T98G

IC_{50} (μM)	48 h	72 h	96 h
Chb	> 50	> 50	> 50
Chb-M'	1.96	0.85	0.34
Chb-S	> 50	25.60	> 50

Supplementary Table 3. IC_{50} of YM155

IC_{50} (nM)	48 h	72 h	96 h
A172	420.3	102.0	66.3
KALS-1	103.0	50.9	51.9

Supplementary Table 4. Target sequences for shRNA[a)]-knockdown experiments

shRNA	Forward (5' → 3')
sh_*RUNX1* #1	AGCTTCACTCTGACCATCA
sh_*RUNX1* #2	AACCTCGAAGACATCGGCA
sh_*BIRC5*	ACGTGTGCTGTCCGT
sh_*Luc*	CGTACGCGGAATACTTCGA

a) shRNA; short hairpin RNA

Supplementary Table 5. PCR and sequencing primer for Sanger sequence

PCR primers	Forward (5' → 3')	Reverse (3' → 5')
TP53 exon 2–4	CAGGAGTGCTTGGGTTGTGG	CGGCATAGGGGGACGTAAAGA
TP53 exon 5–9	TGCCCTGACTTTCAACTCTG	ACCGAGGACCAACATCGATTG
TP53 exon 10–11	ATGCATGTTGCTTTTGTACCG	TATCCACACGCAGTCTTGT

Sequencing primers	Forward (5' → 3')
TP53 exon 6	CTACTGCTCACCCGGAGG
TP53 exon 7	AGGCCTCCCCTGCTTGCC

Supplementary Table 6. PCR primers used for RT-qPCR

PCR primers	Forward (5' → 3')	Reverse (3' → 5')
RUNX1	AGTCATTTCCTTCGTACCCACA	TGGCATCGTGGACGTCTCTA
*RUNX*2	GCCTTCAAGGTGGTAGCCC	AAGGTGAAACTCTTGCCTCGTC
BIRC5	CAGATTTGAATCGCGGGACCC	CCAGAGTCTGGCTCGTTCTCAG
p53	ACAGCACATGACGGAGGTTG	CACACGCAAATTTCCTTCCA
p21	CGACTGTGATGCGCTAATGG	CTCCAGTGGTGTCTCGGTGA
Bcl-xL	GGTTCCCTTTCCTTCCATCC	GGAGTCCTGGTCCTTGCATC
PIF1	CCAGGTGATGCTGGTGAAAA	CTGCCTCGAACCCAACTACC
CHK1	TGGGATACCAGCCCCTCATA	GATCCTGGGGTGCCAAGTAA
GAPDH	CATGTTCGTCATGGGGTGAACCA	AGTGATGGCATGGACTGTGGTCAT

Supplementary Table 7. PCR primers used for ChIP

PCR primers	Forward (5' → 3')	Reverse (3' → 5')
BIRC5	CCAGCCTGGCAAACATGG	CTGCAACCTCCTCCCCGC
PIF1	GAACCTGGACAACTTTCAGTCATC	AAACATTGAACCCAGATTACCTGC
