# Supplementary material for: A RUNX-targeted gene switch-off approach modulates the BIRC5/PIF1-p21 pathway and reduces glioblastoma growth in mice
Source: Commun Biol. 2022 Sep 9;5:939. doi: 10.1038/s42003-022-03917-5 (PMC9463152; doi:10.1038/s42003-022-03917-5)
Supplement: Supplementary file 5 — Reporting Summary [file 42003_2022_3917_MOESM5_ESM.pdf]

nature research

Corresponding author(s): Hiroshi Sugiyama
Yoshiki Arakawa
Yasuhiko Kamikubo

Last updated by author(s): Aug 26, 2022

Reporting Summary

Nature Research wishes to improve the reproducibility of the work that we publish. This form provides structure for consistency and transparency in reporting. For further information on Nature Research policies, see our Editorial Policies and the Editorial Policy Checklist.

Statistics

For all statistical analyses, confirm that the following items are present in the figure legend, table legend, main text, or Methods section.

n/a	Confirmed	
☐	☒	The exact sample size (*n*) for each experimental group/condition, given as a discrete number and unit of measurement
☐	☒	A statement on whether measurements were taken from distinct samples or whether the same sample was measured repeatedly
☐	☒	The statistical test(s) used AND whether they are one- or two-sided<br>*Only common tests should be described solely by name; describe more complex techniques in the Methods section.*
☐	☒	A description of all covariates tested
☐	☒	A description of any assumptions or corrections, such as tests of normality and adjustment for multiple comparisons
☐	☒	A full description of the statistical parameters including central tendency (e.g. means) or other basic estimates (e.g. regression coefficient) AND variation (e.g. standard deviation) or associated estimates of uncertainty (e.g. confidence intervals)
☐	☒	For null hypothesis testing, the test statistic (e.g. *F*, *t*, *r*) with confidence intervals, effect sizes, degrees of freedom and *P* value noted<br>*Give P values as exact values whenever suitable.*
☒	☐	For Bayesian analysis, information on the choice of priors and Markov chain Monte Carlo settings
☒	☐	For hierarchical and complex designs, identification of the appropriate level for tests and full reporting of outcomes
☒	☐	Estimates of effect sizes (e.g. Cohen's *d*, Pearson's *r*), indicating how they were calculated

Our web collection on statistics for biologists contains articles on many of the points above.

Software and code

Policy information about availability of computer code

Data collection	Nothing
Data analysis	Nothing

For manuscripts utilizing custom algorithms or software that are central to the research but not yet described in published literature, software must be made available to editors and reviewers. We strongly encourage code deposition in a community repository (e.g. GitHub). See the Nature Research guidelines for submitting code & software for further information.

Data

Policy information about availability of data

All manuscripts must include a data availability statement. This statement should provide the following information, where applicable:
- Accession codes, unique identifiers, or web links for publicly available datasets
- A list of figures that have associated raw data
- A description of any restrictions on data availability

Our data analysed by microarray were deposited in the Gene Expression Omnibus (GEO) under accession number GSE174634. Its associated figure is Supplementary Figure 5a-c and Supplementary Figure 7e. There are no restrictions on the use of this data.

Field-specific reporting

Please select the one below that is the best fit for your research. If you are not sure, read the appropriate sections before making your selection.

☒ Life sciences ☐ Behavioural & social sciences ☐ Ecological, evolutionary & environmental sciences

For a reference copy of the document with all sections, see nature.com/documents/nr-reporting-summary-flat.pdf

Life sciences study design

All studies must disclose on these points even when the disclosure is negative.

Sample size	Since the differences between the control and target groups were obvious in this experiment, no statistical methods were used to determine sample size. Since reproducibility of at least six times in vivo and three times in vitro is generally required, this number was guaranteed as a minimum. All data were plotted in figure to clarify the results.
Data exclusions	No data were excluded from the analysis.
Replication	Reproducibility of the effect of Chb-M' was obtained by performing the same experiment three times each with WB and RT-PCR for the apoptosis array results. Cells were also created in which the fluctuating genes were repressed or overexpressed, and WB or RT-PCR were used to confirm that the expression trends of the downstream genes were the same as when Chb-M' was administered.
Randomization	All were randomly assigned.
Blinding	Blinding is not relevant to the study because it is not a clinical study that requires group assignment, such as a clinical trial.

Reporting for specific materials, systems and methods

We require information from authors about some types of materials, experimental systems and methods used in many studies. Here, indicate whether each material, system or method listed is relevant to your study. If you are not sure if a list item applies to your research, read the appropriate section before selecting a response.

Materials & experimental systems

n/a	Involved in the study	
☐	☒	Antibodies
☐	☒	Eukaryotic cell lines
☒	☐	Palaeontology and archaeology
☐	☒	Animals and other organisms
☒	☐	Human research participants
☒	☐	Clinical data
☒	☐	Dual use research of concern

Methods

n/a	Involved in the study	
☒	☐	ChIP-seq
☐	☒	Flow cytometry
☒	☐	MRI-based neuroimaging

Antibodies

Antibodies used	anti-RUNX1 (Cat#sc-365644, RRID:AB_10843207; Santa Cruz Biotechnology), anti-RUNX2 (Cat#8486, RRID:AB_10949892; Cell Signaling Technology, Danvers, MA), anti-RUNX3 (Cat#9647, RRID:AB_11217431; Cell Signaling Technology), anti-p21 Waf1/Cip1 (Cat#2946, RRID:AB_2260325; Cell Signaling Technology), anti-survivin (BIRC5) (Cat#GTX100052, RRID:AB_1241366; Gene Tex, Irvine, CA), anti-p53 (Cat#sc-126, RRID:AB_628082; Santa Cruz Biotechnology), anti-PIF1 (Cat#sc-48377, RRID:AB_2164654; Santa Cruz Biotechnology), anti-CHK1 (Cat#sc-8408, RRID:AB_627257; Santa Cruz Biotechnology), anti-Bcl-x (Cat#sc-8392, RRID:AB_626739; Santa Cruz Biotechnology), anti-Bcl-2 (Cat#2872, RRID:AB_10693462; Cell Signaling Technology), anti-BAX (Cat#sc-7480, RRID:AB_626729; Santa Cruz Biotechnology), anti-cyclin A (Cat#sc-271682, RRID:AB_10709300; Santa Cruz Biotechnology), anti-cyclin B1 (Cat#4138, RRID:AB_2072132; Cell Signaling Technology), anti-Cdc25C (Cat#sc-13138, RRID:AB_627227; Santa Cruz Biotechnology) and anti-GAPDH (Cat#sc-47724, RRID:AB_627678; Santa Cruz Biotechnology).
Validation	Validations was performed by each manufactures.

Eukaryotic cell lines

Policy information about cell lines

Cell line source(s)	A172, LN229 and T98G cell lines were purchased from the American Type Culture Collection (ATCC) (Manassas, VA) and KALS-1 were from JCRB cell bank (Osaka, Japan) (A172: ATCC Cat#CRL-1620, RRID:CVCL_0131, KALS-1: JCRB Cat#IFO50434, RRID:CVCL_1323, LN229: ATCC Cat#CRL-2611, RRID:CVCL_0393, T98G: Cat#CRL-1690, RRID:CVCL_0556).

Authentication	ATCC, and STEMCELL Technologies, Vancouver, Canada
Mycoplasma contamination	All cell lines tested negative for myacoplasma.
Commonly misidentified lines (See ICLAC register)	*Name any commonly misidentified cell lines used in the study and provide a rationale for their use.*

Animals and other organisms

Policy information about studies involving animals; ARRIVE guidelines recommended for reporting animal research

Laboratory animals	5-week-old female BALB/cSlc-nu/nu mice (Japan SLC, Shizuoka, Japan) and NOD/Shi-scid, IL-2RγKO Jic (NOG) mice (Kyoto University, Kyoto, Japan)
Wild animals	BALB/cSlc-nu/nu mice, NOG mice
Field-collected samples	brain
Ethics oversight	All animal studies were properly conducted in accordance with the Regulation on Animal Experimentation at Kyoto University, based on International Guiding Principles for Biomedical Research Involving Animals. All procedures employed in this study were approved by Kyoto University Animal Experimentation Committee (permit number: Med Kyo 18293).

Note that full information on the approval of the study protocol must also be provided in the manuscript.

Flow Cytometry

Plots

Confirm that:

- [x] The axis labels state the marker and fluorochrome used (e.g. CD4-FITC).
- [x] The axis scales are clearly visible. Include numbers along axes only for bottom left plot of group (a 'group' is an analysis of identical markers).
- [x] All plots are contour plots with outliers or pseudocolor plots.
- [x] A numerical value for number of cells or percentage (with statistics) is provided.

Methodology

Sample preparation	Apoptosis assay was performed by APC Annexin V Apoptosis Detection Kit with PI (BioLegend, San Diego, CA). In brief, approximately 1 ×10^5 cells of the indicated control and experimental groups were washed twice with phosphate-buffered saline (PBS) and suspended in annexin-binding buffer. Next, 5 µL of annexin V and 10 µL of propidium iodide solution (BioLegend) was added. Reaction mixtures were incubated at 25 degrees for 15 min, and cells were processed for flow cytometric analysis.
Instrument	FACS Canto II, BD (Franklin Lakes, NJ)
Software	FlowJo (BD) was used. Accessed using a USB type license key.
Cell population abundance	After sort, debris was removed using FSC and SSC based gates, then doublet treatment was performed by plotting Height and Wide with FSC and SSC. And all live and dead cells were counted.
Gating strategy	After removing debris using FSC and SSC gates, doublet processing was performed by plotting Height and Wide in FSC and SSC.<br>Annexin V+/propidium iodide (PI)- cells and Annexin V-/PI+ cells were used to threshold the vertical (PI) and horizontal (Annexin V) axes, respectively.

- [x] Tick this box to confirm that a figure exemplifying the gating strategy is provided in the Supplementary Information.
